# Mechanisms and advances in field assisted machining of SiC

**DOI:** 10.1007/s00170-026-17695-5

**Published:** 2026-03-14

**Authors:** Peixiong Wang, Wenbin Zhong, Maomao Wang, Haodong Zhao, Wenrui Wu, Peixuan Yan, Wenhan Zeng, Xiangqian Jiang

**Affiliations:** Centre for Precision Technologies, School of Computing and Engineering, University of Huddersfield, Huddersfield, West Yorkshire UK

**Keywords:** Field-assisted machining, Hard and brittle materials, SiC, Hardness

## Abstract

Silicon carbide (SiC) has become an increasingly important material for optical and semiconductor applications owing to its exceptional properties. However, its extreme hardness and brittleness pose significant challenges to traditional machining processes, leading to severe tool wear, low material removal rates, and suboptimal surface finishes. To overcome these issues, advanced field-assisted techniques like laser-assisted machining have emerged as promising solutions. This review provides a comprehensive overview of the mechanisms and recent advances in field-assisted machining processes of SiC, with a particular emphasis on laser-assisted machining. Critical research gaps are identified and a potential processing roadmap is outlined to pave the way for high-quality and efficient machining of SiC.

## Introduction

**Fig. 1:**
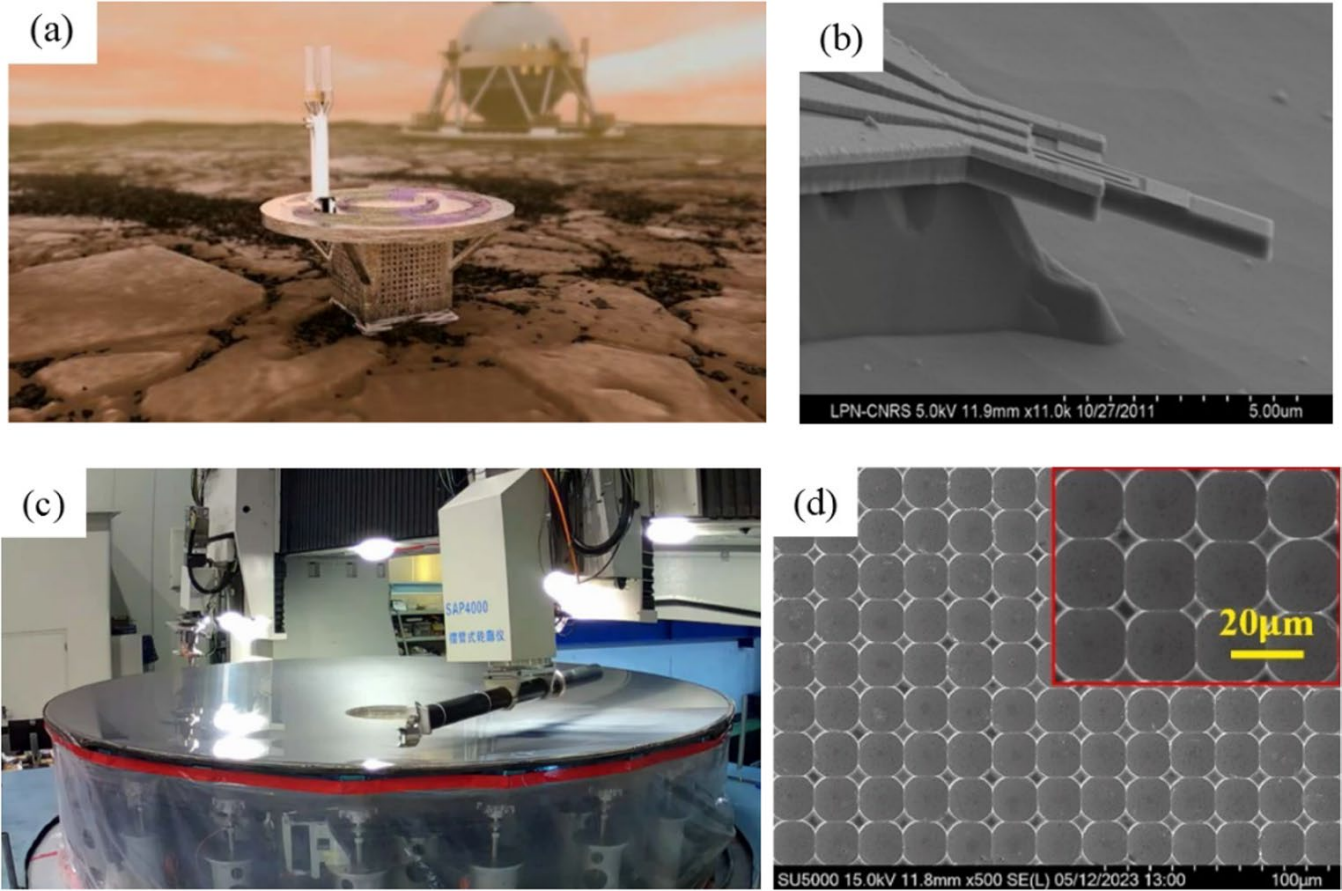
Applications of SiC. (**a**) Sustainable operation of SiC integrated circuits in a Venus-simulated environment. (**b**) The MEMS nanocantilever for atomic force microscopy. (**c**) Large-aperture SiC mirrors used in telescope systems. (**d**) Microlens arrays used in optoelectronic devices [2–4]

Driven by the rapid development of industries such as optical instruments, controllers, and wireless communication, the demand for high-performance and reliable power semiconductor devices has intensified. Hard and brittle materials (HBMs) have emerged as indispensable materials in modern optics and semiconductor fields, attributed to their unique physical, mechanical, and optical properties. As shown in Fig. 1, with advancements in processing technologies, the applications of HBMs have become widely expanded. As a representative HBM, silicon carbide (SiC) has been increasingly utilized across various fields due to its exceptional properties [1].

SiC-based materials can be categorized into two main types: single-crystal SiC and ceramic SiC. Within single-crystal SiC, owing to the diversity of crystal structures, the most common variants are 4 H-SiC and 6 H-SiC, which exhibit α-SiC characteristics, as well as 3 C-SiC, which exhibits β-SiCs characteristics [5]. By contrast, silicon carbide ceramics are often manufactured as reaction-bonded silicon carbide (RB-SiC), which can also serve as the matrix for SiC-based ceramic-matrix composites via reactive melt infiltration, such as C/SiC and SiC_f_/SiC [6]. As shown in Table 1, in terms of mechanical properties, SiC exhibits a Mohs hardness of up to 9, providing exceptional wear resistance [7]. Additionally, SiC has a melting point as high as 2830 °C, which ensures excellent thermal stability under extreme conditions and enables its application in high-temperature environments [8]. Moreover, its low thermal expansion coefficient and high thermal conductivity maintain structural integrity and heat dissipation efficiency under rapid temperature fluctuations, making it suitable for aerospace and high-temperature manufacturing applications [9–12]. In terms of optical properties, SiC has a refractive index of 2.65, offering excellent optical transparency and enabling its use in broad-spectrum optical applications [13]. Its wide bandgap further ensures optical stability, allowing SiC to maintain superior performance even in radiation-rich environments, which makes it a ideal selection for deep-space exploration, laser systems, and infrared windows [14–16]. Regarding electrical properties, SiC exhibits high electron mobility and saturation drift velocity, providing a solid foundation for its application in high-frequency and high-power electronic devices [17]. Its high breakdown electric field and wide bandgap characteristics make it well-suited for high-power electronics operating in high-temperature environments. Moreover, SiC’s chemical stability and corrosion resistance allow it to maintain performance in strong acids, strong alkalis, and other harsh chemical environments. Consequently, with superior performance and higher stability compared with conventional Si-based materials, SiC is increasingly recognized as an irreplaceable core material in optical, electronic, and mechanical applications.

**Table 1:** The properties of SiC at 300 K [18–20]

Property	3 C-SiC	6 H-SiC	4 H-SiC
CAS Registry Number	409-21−2	409-21−2	409-21−2
Molecular Weight	40.097	40.097	40.097
Average atomic weight	20.1	20.1	20.1
Melting Point	2830℃	2830℃	2830℃
Density	3.16 g/cm⁻³	3.16 g/cm⁻³	3.16 g/cm⁻³
Solubility in water	Insoluble	Insoluble	Insoluble
Solubility in ethanol	Insoluble	Insoluble	Insoluble
Dielectric Tensor Components	9.72	9.66	9.7
Hardness	9 Mohs	9 Mohs	9 Mohs
Coefficient of thermal linear expansion	2.77 × 10⁻⁶K⁻¹	<=2.4 × 10⁻⁶K⁻¹	<=2.4 × 10⁻⁶K⁻¹
Thermal conductivity	3.2 W/(cm·K)	4.9 W/(cm·K)	3.7 W/(cm·K)
Index of refraction	2.65	2.65	2.65
Band gap	2.3 eV	3.0 eV	3.2 eV
Electron mobility	750–800 cm^2^/V·s	60–400 cm^2^/V·s	800 cm^2^/V·s
Hole mobility	40 cm^2^/V·s	90–100 cm^2^/V·s	115 cm^2^/V·s
Saturated electron drift velocity	2.5·10^7^ cm/s	2.0·10^7^ cm/s	2.0·10^7^ cm/s
Breakdown field	1.8 MV/cm	1–3.2.2 MV/cm	2.5–3.0.5.0 MV/cm
Physical stability	Excellent	Excellent	Excellent

**Fig. 2:**
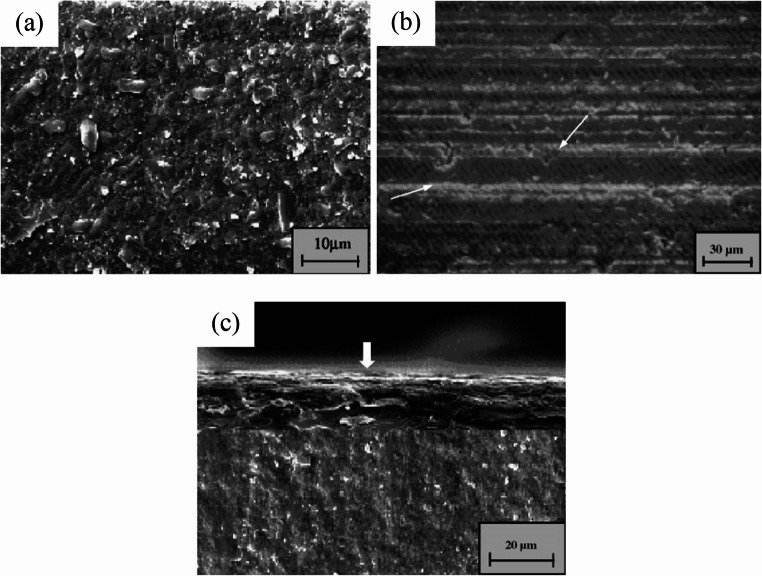
(**a**) Microstructure of SiC (**b**) Surface brittle fractures of SiC during mechanical machining (**c**) Subsurface damage of SiC during mechanical machining. The arrow indicates the machined surface [22]

However, as a typical HBM, SiC still presents numerous challenges to machining. As shown in Fig. 2, owing to its high brittleness, significant surface and subsurface damage, such as scratches and microcracks, often occurs during grinding and mechanical polishing [21]. These defects not only degrade the surface quality of the machined workpiece but also impair its mechanical and optical properties.

A variety of field-assisted techniques are currently used for machining HBMs. Among them, laser-assisted techniques have attached the most attention and regarded as one of the most promising approaches for machining HBMs [23]. The remainder of this paper is organized as follows. Sect. 2 reviews the current research status and identifies the key machining difficulties of SiC. Sect. 3 surveys field-assisted machining techniques which may address these challenges for SiC machining. Sect. 4 focuses on the current research status on laser-assisted diamond turning of SiC. Sect. 5 summarizes advances in SiC surface modification with pulsed lasers. Finally, we propose potential future directions aimed at addressing the existing challenges and advancing the understanding and application of laser-assisted cutting in the precision machining of SiC.

## Current research status of SiC machining

Currently, the machining of SiC typically involves an initial grinding step, followed by further refinement using chemical mechanical polishing (CMP) [24, 25]. However, owing to the high chemical stability of SiC, its reaction rate with chemical reagents is relatively slow. This makes it difficult to improve the efficiency of this process [26]. In addition, the increasing demand for the machining of freeform surface in integrated optical components poses further challenges. The conventional combination of grinding and CMP is limited in its ability to simultaneously achieve both high precision and freeform surface machining, and consequently fails to meet the technical requirements of modern ultra-precision manufacturing.

Single point diamond turning (SPDT) is an advanced ultra-precision machining technology that is used to fabricate optical components by removing minute amounts of material with extremely high precision. In SPDT, material removal from the workpiece is achieved solely by mechanical cutting. Single-crystal diamond, as one of the hardest known material, is used to produce cutting tools with nanoscale edge radii, enabling efficient and stable cutting operations [27]. By precisely controlling the relative position of the tool and the workpiece through computer numerical control systems, SPDT can produce complex optical components with high precision in a single setup. After years of research and exploration, SPDT has become one of the most effective and economical methods for optical component manufacturing.

**Fig. 3:**
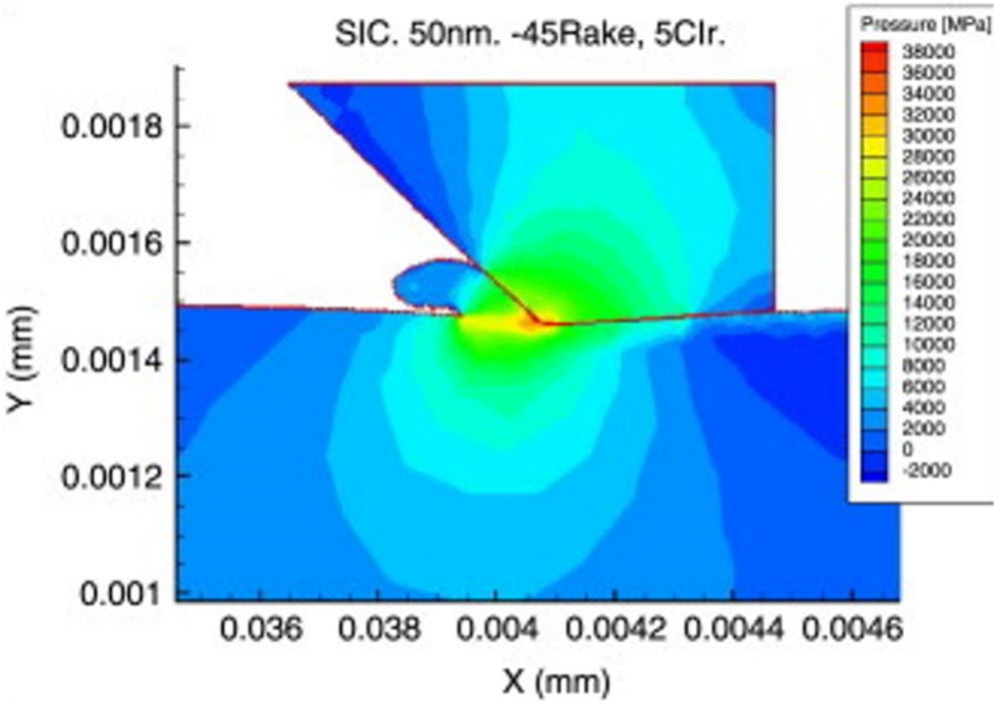
Simulation of machining pressure at a cutting depth of 50 nm [29]

Patten [28] first proposed that the brittle-to-ductile transition (BDT) in SPDT of SiC is caused by the formation of a high-pressure phase at the cutting edge, based on observations of the surface morphology and chip composition of machined 6 H-SiC. This high-pressure phase transformation mechanism allows SiC surfaces within a localized region to undergo plastic deformation instead of brittle fracture, laying the groundwork for subsequent studies on the SPDT machining mechanism of SiC. Later, Patten [29] utilized the pressure-sensitive Drucker–Prager constitutive model proposed by Ajjarapu [30] to simulate the machining process using 2D turning analysis. These simulations investigated cutting forces, thrust forces, and the BDT behaviour under different cutting depths and rake angle. The results demonstrated that the model accurately predicts forces under ductile conditions, closely matching experimental results obtained at shallow cutting depths. The BDT occurs below a critical cutting depth. As shown in Fig. 3, when the cutting depth is 50 nm, the primary removal mechanism in SPDT of SiC is ductile mode cutting.

**Fig. 4:**
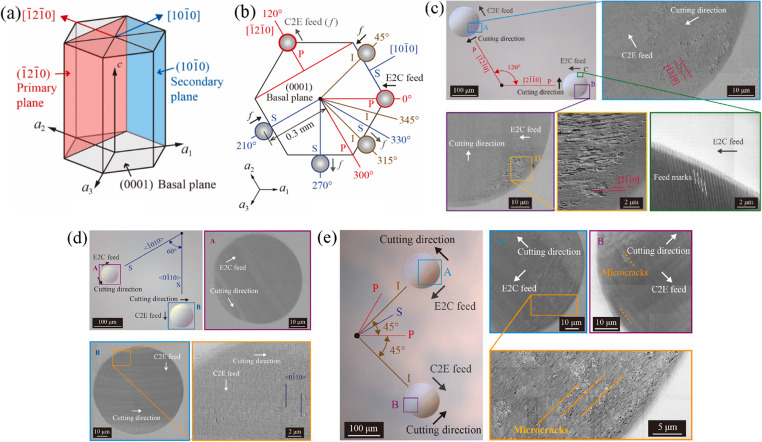
(**a**) Crystal structure of 4 H-SiC (**b**) Machining directions along < − 12 − 10> and < 10–10> (**c**) Crack morphology in the < − 12 − 10> machining direction (**d**) Crack morphology in the < 10–10 > machining direction (**e**) Crack morphology in the intermediate direction between < − 12 − 10> and < 10–10> [34]

Yan [31] conducted SPDT on RB-SiC using diamond tools with different rake angles and tool nose radii. The results showed that material removal is dominated by the plastic deformation of 6 H-SiC grains and the amorphization-induced displacement of Si-C bonds, with no evidence of large-scale fracture. Tool wear manifested primarily as micro-chipping in tools with zero rake angle and tools with highly negative rake angles exhibited micro-grooved flank. At high feed rates, tools with a −40° rake angle reduced micro-cracks and tool wear compared with tools of 0° rake angles. Goel [32], building upon the ductile machining model proposed by Scattergood and Blake [33], investigated the microscopic behaviour and tool wear characteristics during 6 H-SiC machining. The results indicated that material removal at short distances and shallow cutting depths primarily occurs through plastic deformation. For single-crystal 6 H-SiC, chip formation is achieved through lattice distortion, whereas polycrystalline SiC undergoes grain sliding, leading to stress accumulation at grain boundaries. Consequently, material removal in polycrystalline SiC is influenced by grain-boundary phase transitions and grain sliding, giving single-crystal SiC an advantage in achieving high-quality surfaces. Under distilled water coolant, high-quality surfaces can be produced at short cutting distances for 6 H-SiC. However, at long cutting distances, significantly increased cutting resistance leads to pronounced tool wear and subsequently degraded surface quality. Thus, short tool life and limited machining range remain major challenges in SiC machining. To further improve tool life, Huang [34] studied the influence of different crystal orientations in the machining performance of single-crystal 4 H-SiC during ultra-precision diamond turning. The interaction mechanisms between the diamond tool and SiC were also examined. The results revealed that SiC can exhibit ductile mode cutting. However, under high cutting forces, surface cracks are more likely to form in feed-in and cut-in region. As shown in Fig. 4, machining in the < − 12 − 10> crystal direction (P direction) can better suppress the formation of surface cracks, although subsurface damage still cannot be completely eliminated. Cutting along the < 10–10 > direction results in not only surface cracks but also severe subsurface damage. Machining along intermediate crystal orientations reduces the depth of the subsurface damage layer but increases the likelihood of surface cracking. These surface cracks are primarily caused by tensile stresses generated along the cutting direction due to tool-workpiece friction.

Through years of sustained efforts and research, SPDT has achieved considerably in the ultra-precision machining of SiC, with surface roughness has decreased and machining accuracy has shown remarkable improvements. Although SPDT can process SiC by BDT mechanism, it still faces numerous unresolved challenges in SiC machining. During machining, SiC leads to severe tool wear and shortened tool lifespan. Additionally, its low material removal rate results in prolonged machining times and increased energy consumption. These factors not only increase processing costs but also reduce processing efficiency. Furthermore, surface cracks and subsurface damage generated during machining are still difficult to eliminate completely. In applications requiring high precision and low defects, the current SPDT technology is still unable to meet the stringent demands for nanoscale surface quality. Relying solely on conventional single-point diamond turning techniques is insufficient to achieve further breakthroughs in SiC machining. Therefore, new methods and technological approaches must be explored.

## Overview of field-assisted machining techniques

To address the limitations of conventional machining techniques in machining HBMs, the introduction of external energy fields into traditional mechanical manufacturing processes, known as field-assisted cutting (FAC), has increasingly attracted the attention of researchers. As an advanced manufacturing technique, FAC can effectively improve machining conditions, reduce cutting forces, and enhance the machinability and surface quality of HBMs. Depending on the mode of energy field application, FAC can be categorized into three types. The first mode involves applying the energy field to the cutting tool, such as applying ultrasonic vibration on the tool [35].The second mode applies the energy field directly to the workpiece surface, enhancing machinability through surface softening or modification, utilizing methods such as laser treatment, ion implantation, and magnetization [36–38]. The third mode combines the two methods, applying different energy fields to both the workpiece and the tool simultaneously during machining, further enhancing machining performance [39, 40].

### Ultrasonic vibration assisted machining (UVAM)

**Fig. 5:**
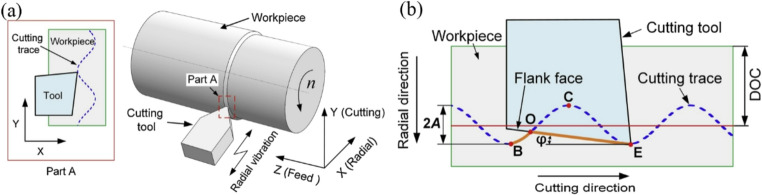
Ultrasonic vibration-assisted machining. (**a**) Process schematic and (**b**) Tool path diagram [44]

UVAM can apply high-frequency, low-amplitude vibrations to the cutting tool or workpiece. The fundamental principle is that altering the contact characteristics of the conventional cutting process through high-frequency vibrations, transforming continuous cutting into periodic, discontinuous cutting. This significantly reduces cutting forces, improves surface quality, and extends tool life. UVAM was initially developed for machining metals, and was subsequently extended to drilling, milling, grinding, and other conventional machining techniques, gradually evolving into various vibration modes such as one-dimensional vibration, two-dimensional elliptical vibration, and three-dimensional compound vibration [41–43]. A schematic diagram of UVAM is shown in Fig. 5.

The mechanisms of UVAM primarily consists of intermittent cutting and the material softening effect [45]. Intermittent cutting results from the periodic separation between the tool and the workpiece caused by high-frequency vibrations, which reduces cutting forces and friction. The material softening effect results from local temperature rise induced by ultrasonic vibrations, leading to a reduction in surface hardness of the workpiece. This enhances the material’s plastic deformation capability and reduces cutting resistance.

Zhao [46] applied ultrasonic vibration-assisted polishing for finishing of linear micro-cylindrical grooves of SiC and developed a friction–normal force model incorporating a corrected Hertzian contact pressure. Experiments showed that, at vibration frequencies of 25 and 35 kHz with micrometer-scale amplitudes, the application of UVAM significantly reduced the interfacial friction force. Analysis of the Stribeck curve indicated that the lubrication state still fell within the boundary-lubrication regime, but shifted closer to film lubrication regime. XRD measurements further revealed the formation of a soft SiO₂ reaction layer in the polishing slurry, which participates in the material removal process and thus indicates a chemo–mechanical synergistic mechanism. By using a combination of low load, low feed rate, and high-frequency, high-amplitude excitation, the surface roughness of the micro-cylindrical groove can be reduced from approximately 109 nm after grinding to about 4 nm. This strong dependence on a specific combination of load, feed and vibration parameters also implies that the effective process window of UVAM is relatively narrow. Ban [47] developed a molecular dynamics model for ultrasonic vibration-assisted single-abrasive-grain scratching of 4 H-SiC. The results indicate that friction force decreases with increasing amplitude and frequency, with amplitude having the dominant effect. Temperature rises with increasing amplitude, whereas its dependence on frequency exhibits a decrease followed by increase trend. Introducing ultrasonic vibration during processing promotes the formation of longitudinal dislocation formation, thereby facilitating material removal. A combination of high-amplitude and high-frequency markedly enhances material-removal efficiency. A low-amplitude with high-frequency combination produces smooth grooves and suppresses lateral pile-up on both sides of the scratch, whereas a low-amplitude with low-frequency combination more effectively suppresses transverse dislocations and subsurface damage, improving machining precision. Yu [48] conducted three dimensional finite element modelling of a SiC ceramic-matrix material in Abaqus, complemented by ultrasonic milling experiments. The results show that feed-direction ultrasonic vibration narrows the contact stress zone, reduces peak of cutting force, and reduces damage and surface defects in both micro brittle fracture regime and macro brittle fracture regime, yielding a lower surface roughness. By contrast, ultrasonic vibration perpendicular to feed direction increases the effective cutting depth in the micro brittle fracture regime and thereby degrades surface flatness. However, in the macro brittle fracture regime the tension compression cycles promotes brittle fracture of fibres and chip evacuation, leading to lower force peaks and a shallower damaged layer.

However, UVAM still has some limitations in machining. Chen [49] performed molecular dynamic simulations of 3 C-SiC machining. The results demonstrate that in the low-frequency region UVAM can exacerbate taper slips and subsurface dislocations in 3 C-SiC. Increasing the vibration amplitude significantly increases the material removal rate but leads to an approximately linear increase in surface roughness. Liu [50] conducted an experimental investigation into the tool wear behaviour in conventional turning, ultrasonic vibration-assisted turning and water-cooled ultrasonic vibration-assisted turning of SiC_f_/SiC ceramic matrix composites. The results showed that the combined use of ultrasonic vibration and water cooling can reduce cutting forces, tool wear, and surface roughness, although tool wear remained significant. Analysis of worn tool topographies reveals that flank wear, spalling of polycrystalline diamond grains and edge chipping are the dominant wear patterns, which are closely related to the extremely high hardness of SiC_f_/SiC and the high-frequency impact introduced by radial ultrasonic vibration. The debris generated during machining which composed of hard SiC fibres, SiC matrix fragments and even partially spalled diamond grains, tends to accumulate on the rake and flank faces, where it acts as secondary abrasives and aggravating tool wear.Radial ultrasonic vibration-assisted turning and water cooling can remove part of these debris and reduce the local thermal–mechanical load. However, they still unable to prevent progressive tool degradation.

In summary, due to the fact that HBMs combine very high hardness with pronounced brittleness, introducing ultrasonic vibration tends to intensify intermittent impacts between the tool and workpiece. This impact loading accelerates fatigue in the tool and vibration components. At the same time, although UVAM reduces cutting forces, the localised stresses at the cutting edge remains high under high-frequency, intermittent contact, promoting accelerated wear, edge chipping and surface and subsurface defects. Consequently, the large-scale industrial use of UVAM for HBMs is still limited.

### Magnetic-assisted machining (MAM)

MAM can enhance machining performance by superimposing a magnetic field on conventional mechanical machining processes. This method improves machining efficiency and stability by influencing material deformation, cutting forces, tool vibrations characteristics, and surface quality. MAM can be classified into two types: static magnetic field machining and dynamic magnetic field machining. Static magnetic field machining applies a magnetic field generated by permanent magnets to the cutting zone. Dynamic magnetic field machining uses electromagnetic devices to dynamically control the magnetic field. During cutting, the magneto plastic effect induced by the magnetic field facilitates dislocation depinning, reducing the material’s yield strength and enhancing its plastic deformability. This effect is particularly evident in nonmagnetic or weakly magnetic materials such as single-crystal copper and nickel-based superalloys, thereby improving their machinability under magnetic-assisted machining [51]. Additionally, under magnetic field influence, the tool surface may form a microscopic magnetized layer or undergo minor surface restructuring within localized contact areas. This helps establish a more stable contact state, reduces adhesive wear, and improves friction characteristics between the tool and the workpiece [52]. Consequently, the application of a magnetic field can reduce tool wear, optimize cutting force distribution, and extend tool life. In the presence of a magnetic field, especially under dynamic or high-intensity magnetic field conditions, Lorentz forces are induced within the cutting zone. These Lorentz forces stabilize chip formation and flow, reducing irregular chip fragmentation and promoting more continuous chip formation. Furthermore, the damping effect produced by Lorentz forces can suppress high-frequency tool vibrations in the cutting zone, thereby reducing tool wear.

El Mansori [53] conducted dry cutting experiments on AISI 1045 carbon steel using non-magnetic carbide tools and an externally applied dc magnetic field. The study found that the introduction of a magnetic field modified the friction characteristics in the tool-chip contact zone. Under the influence of the magnetic field, a continuously replaced built-up edge was formed, which reduced the friction coefficient and tool wear. The application of the magnetic field significantly enhanced tool wear resistance, leading to a notable extension in tool life, particularly under high field intensities. Khalil [54] investigated the effects of magnetic fields on the machining performance of Ti_6_Al_4_V using a self-designed magnetic-assisted SPDT system. The results showed that the magnetic field significantly enhanced cutting performance by reducing heat accumulation and built-up edge formation. At a magnetic field strength of 0.02 T, the surface roughness of the workpiece was reduced by 33%, reaching 13.33 nm. Meanwhile, tool wear was significantly reduced, and chip morphology became more continuous and uniform. Li [55] studied the mechanisms of magnetic-assisted SPDT in machining titanium alloys and its effect on vibration suppression. The results indicated that the external magnetic field introduced eddy-current damping forces during the cutting process, suppressing self-excited vibrations in the tool-workpiece system. This reduced surface roughness by approximately 39%, reaching 16.1 nm. At the same time, the thermal conductivity of the titanium alloy was improved, reducing heat accumulation in the cutting zone.

However, due to its strong covalent bonding and the absence of unpaired electrons, SiC is non-magnetic and cannot generate significant magneto plastic effects or eddy current damping effects in an applied magnetic field. As a result, the influence of magnetic fields on dislocation motion in SiC is weak. An external magnetic field cannot effectively reduce cutting resistance or improve plastic deformability. In addition, the electromagnetic interference and increased system complexity associated with magnetic field devices pose challenges for controlling machining precision. Therefore, the practical application of magnetic-assisted cutting in processing SiC still requires further optimization and breakthroughs.

### Ion implantation-assisted machining (IIAM)

**Fig. 6:**
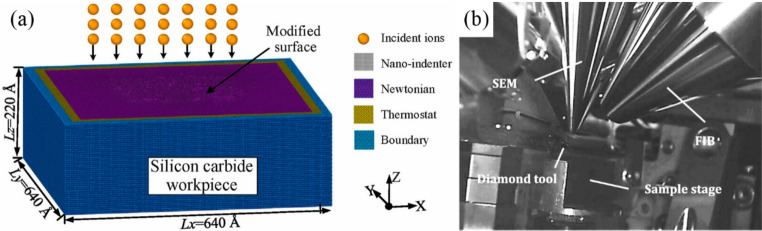
Schematic diagram of ion implantation surface modification [56, 57]

Ion implantation-assisted machining technology irradiates the material surface with an ion beam to form a modified layer, thereby improving the machinability of the material. This technology significantly outperforms traditional mechanical machining methods in terms of reducing machining damage, lowering cutting forces and tool wear, and improving surface quality. The machining mechanism of this technology utilizes high-energy ions to bombard the workpiece. As show in Fig. 6, during this process, high-energy ions transfer energy to surface atoms through elastic and collisions. This energy transfer disrupts the lattice integrity of the target material, forming an amorphous layer or a region with high-density defects. This amorphous structure reduces hardness and brittleness of the material, enhances brittle-to-ductile transition depth, and increases the plastic machining depth of HBMs. At the same time, it also reduces cutting forces and tool wear during machining.

**Fig. 7:**
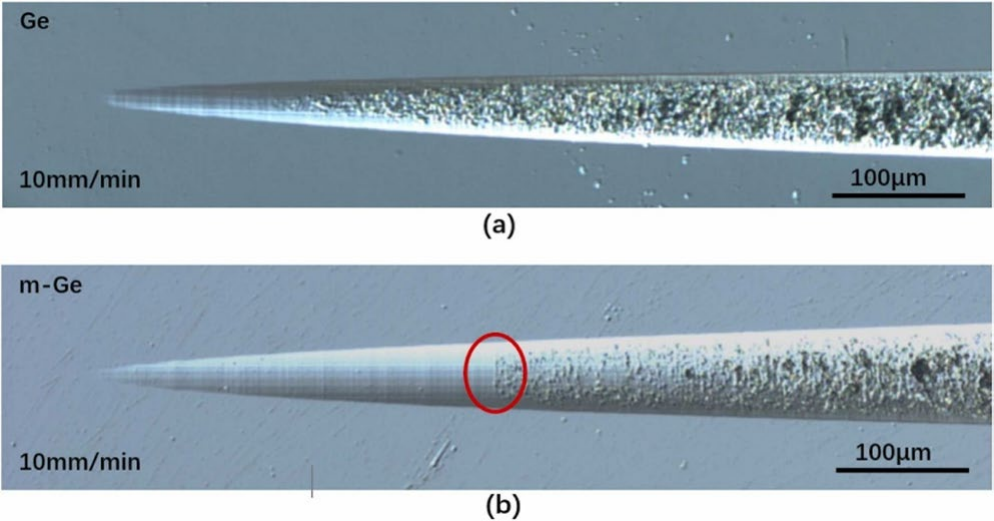
Monocrystalline germanium scratch test micrographs. (**a**) before modification (**b**) after modification [60]

In recent years, many researchers have investigated various aspects of ion implantation-assisted machining. Fan [58] used the stopping and range of ions in matter method to conduct molecular dynamics simulations of Ga ion implantation and cutting on single-crystal 4 H-SiC at various implantation doses. The results showed that as the gallium ion implantation dose increased, the surface amorphization of 4 H-SiC increased significantly. Its lattice structure gradually transformed from ordered to disordered. During machining, cutting forces and stress fluctuations were notably reduced, while material ductility and removal efficiency improved. Additionally, the amorphous layer formed by ion implantation effectively alleviated stress concentrations in the machining region, reducing tool wear and machining instability, thereby enhancing surface quality. Dai [59] employed molecular dynamics simulations to study the surface modification and machining performance of 3 C-SiC after silicon ion implantation at different energy levels. The results indicated that ion implantation can significantly alter the physical properties of the material surface, forming a modified layer with an amorphous structure. As the implantation energy increased, the system potential energy also increased, leading to reduced cutting forces, slower temperature rise rates, and significantly fewer dislocations and residual defects during machining. Furthermore, higher implantation energy resulted in a thicker amorphous layer and more stable cutting behaviour. However, when the energy exceeded 1 keV, surface damage increased significantly. As the critical cutting depth increased, the proportion of plastic removal gradually rose, and surface roughness was notably reduced. Although high implantation energy introduce more surface damage it enhance the proportion of plastic removal and thereby the machinability of 3 C-SiC. Tan [60] simulated the distribution and displacement of implanted ions and experimentally verified the resulting surface modification in monocrystalline germanium using 200 keV hydrogen ions, 200 keV helium ions, and 400 keV copper ions. The results showed that copper ions formed an amorphous layer about 300 nm in thickness on the surface, while helium and hydrogen ions modified the middle depth and deeper subsurface regions, respectively. As shown in Fig. 7, in scratch tests, the ductile removal region of ion-implanted monocrystalline germanium was significantly extended compared to unmodified samples. For the modified monocrystalline germanium, the brittle-to-ductile transition depth increased by over 200 nm at all tested machining speeds.

Although ion implantation-assisted machining has demonstrated considerable advantages in improving the machinability of SiC, it still faces several critical constraints. Ion implantation can induce material damage such as deep surface cracks and excessive amorphization. Moreover, ion implantation equipment is expensive, and the process requires a vacuum environment, resulting in high processing costs. This issue is particularly significant for multiple implantations or high-dose implantation processes, making large-scale industrial applications of ion implantation-assisted machining challenging.

### Laser-assisted machining (LAM)

Laser-assisted machining irradiates the workpiece surface with high-energy-density laser beam, causing localized melting, evaporation, or even sublimation, as well as photothermal or photochemical reactions. It offers the characteristics of high energy density, high precision, high efficiency, and non-contact processing [61]. Additionally, the laser beam can be precisely directed and focused into a tiny focal spot to generate a highly localized heat affected zone [62]. Accordingly, LAM has been widely adopted in precision machining.

**Fig. 8:**
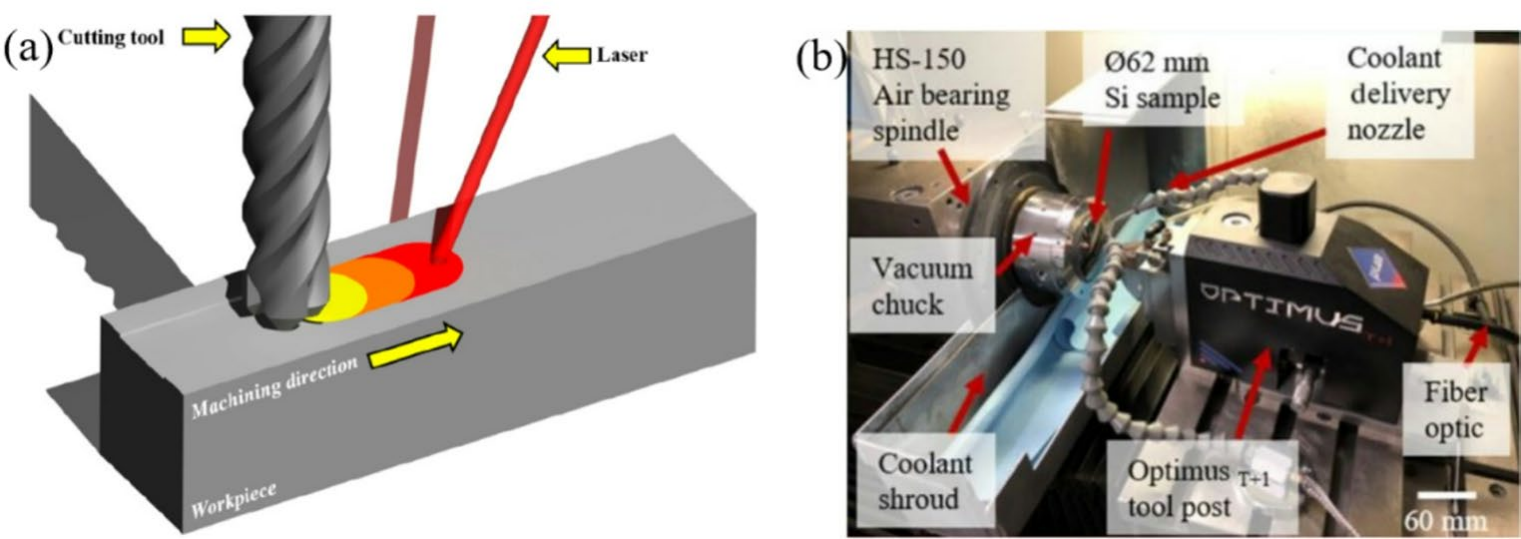
Laser-assisted machining system. (**a**) Laser-assisted milling [68], (**b**) Laser assisted diamond turning system [69]

Lasers are mainly classified into pulse lasers and continuous lasers according to their working conditions. Continuous-wave laser deliver energy continuously and typically feature a relatively large spot size. Its heating effect on materials is temporally stable and continuous, making continuous-wave lasers widely used in thermal processing and material modification [63]. Pulse laser are characterized by extremely high instantaneous power and are typically used for material cutting, removal and surface modification [64]. In particular, ultra-short pulse lasers exhibit minimal thermal effects and a extremely small heat-affected zone during surface modification processes, which helps to avoid thermal deformation, micro-cracks, or structural changes caused by excessive heat input. Owing to their intrinsic high hardness and pronounced brittleness, HBMs are difficult to machine using traditional mechanical methods [65]. As a non-contact machining method, laser processing enables effective material removal or surface modification without considering the wear on the processing tools [66]. After laser pretreatment of the surface of certain materials, the mechanical properties of the material surface can be significantly changed [67]. This modification is conducive to the selection of parameters in the subsequent mechanical processing process, can effectively reduce the tool wear, improve the surface quality obtained by processing, and increase processing efficiency. The LAM system is shown in Fig. 8.

Currently, there are two primary laser-assisted machining strategies used for HBMs. The first approach integrates the laser with the machining centre, where the laser elevates the surface temperature of the workpiece locally and instantaneously, resulting in thermal softening. The cutting tool then removes the softened layer in real time. The second approach utilizes laser preprocessing, in which the laser pre-removes part of the surface material and induces microcracks, phase transformations, and localized surface or subsurface damage. This laser-induced weakened layer significantly reduces the surface strength of the material, after which the workpiece is subsequently machined using conventional tools [70, 71].

**Fig. 9:**
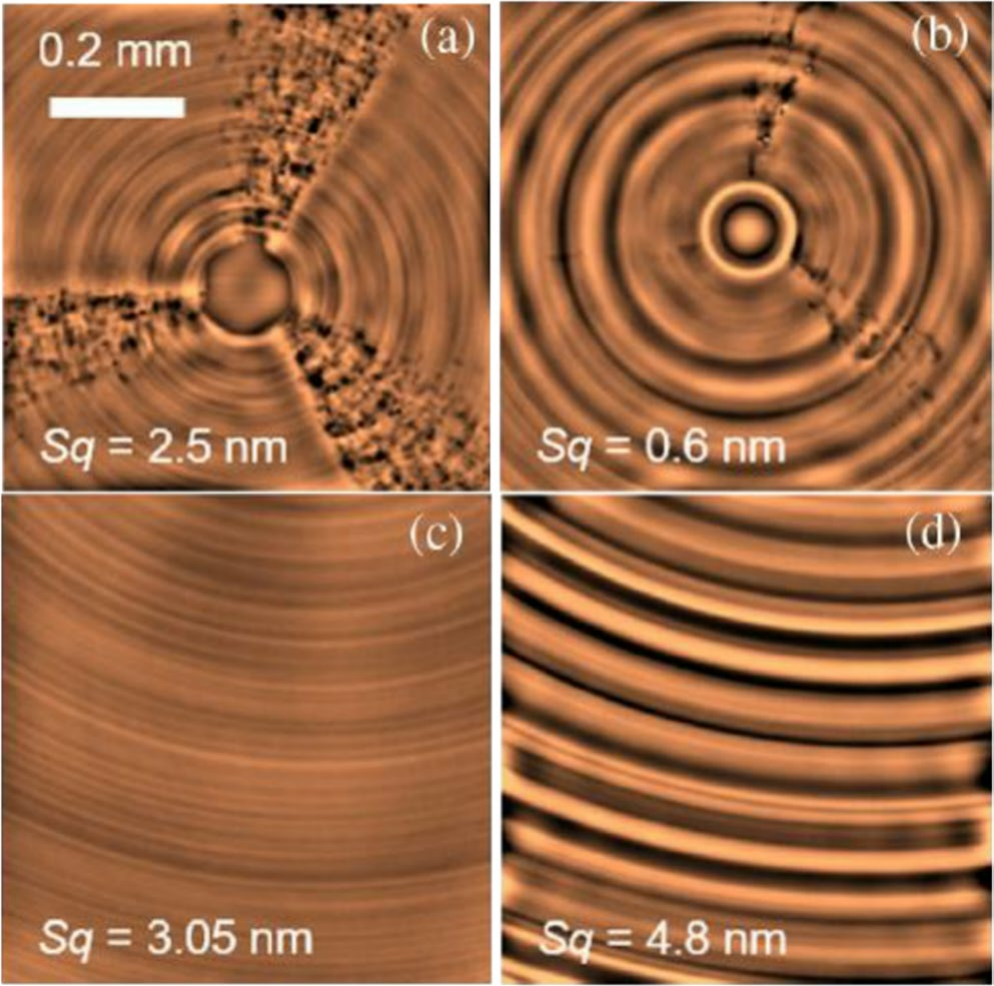
Surface morphology of single crystal silicon wafer after processing [69]. (**a**) Traditional diamond turning centre area. (**b**) Laser-assisted turning centre area. (**c**) Traditional diamond turning outer ring area (**d**) Laser-assisted turning outer ring area

**Fig. 10:**
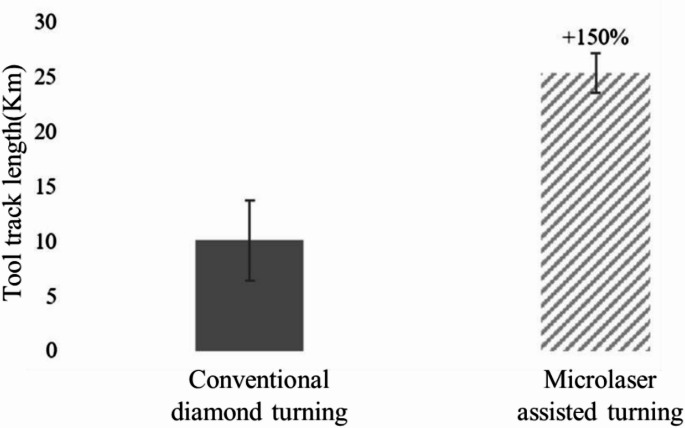
Life of diamond tools for cutting single crystal silicon [69]

Kumar [72] used a fibre laser at a power of 15 W and at a wavelength of 1.06 μm to scan the ceramic surface, thereby inducing localized thermal stresses within the material. During the real time removal of the heat affected zones using micro-grinding rods, the maximum grinding force was reduced by 43.2%, and the material removal rate was correspondingly enhanced. Chih [73] built a specialized laser-assisted planning system using a laser source and a mechanical planer. The Al_2_O_3_ ceramic material was subsequently planed by the mechanical tool immediately following laser ablation. Under laser irradiation, the surface temperature of the material rapidly rises above the glass transition temperature, making the softened alumina ceramic more amenable to planning. During the machining process, the cutting force was reduced by approximately 10–16%, whereas the surface roughness was increased by about 74%. Shahinian [69] developed the micro laser-assisted machining system(µ-LAM) to enable ultra-precision processing of single-crystal silicon. In this system, a continuous laser is positioned behind the diamond turning tool holder and operates in conjunction with a dedicated cutting zone cooling system. During machining, the laser can provide a stable local heating effect at the cutting point, thereby the overall temperature rise of the workpiece and turning tool can be controlled, and the impact on surface shape errors and tool wear can be reduced. As shown in Fig. 9, The surface roughness of the machined workpiece reaches the nanometre level, and the influence of single crystal silicon anisotropy on the machining quality is markedly mitigated. A comparative assessment of tool wear between conventional diamond turning and laser-assisted turning was conducted under identical machining conditions. As shown in Fig. 10, laser-assisted turning can effectively reduce tool wear, resulting in a 150% increase in tool life.

As summarized in Table 2, LAM demonstrates the most favourable overall performance among the four field-assisted routes. The localized thermal input induces material softening and phase transformation, thereby promoting the brittle-to-ductile transition, which in turn produces the greatest reduction in cutting forces and establishes the most stable tool–workpiece contact state. The resulting enhancement enables the use of larger depth of cut and higher feed rate, leading to a substantial increase in material-removal rate and overall machining throughput. In terms of surface integrity, LAM markedly reduces surface roughness while significantly reducing subsurface brittle-crack formation. The heat affected zone, oxidation, and recast layer formation associated with LAM can be effectively controlled by gas-jet cooling and pulsed laser operation, further complemented by low-heat-input scanning strategies [74, 75]. These findings indicate that applying laser-assisted processing to HBMs can significantly improve the surface quality of HBMs components. Compared with conventional manufacture, LAM also offers substantial advantages in reducing cutting force, extending tool life, and improving both machining quality and overall processing efficiency [76].

**Table 2:** Field-assisted processing comparison

Dimensions	LAM	UVAM	MAM	IIAM
Core mechanism	Local heating produces softening or phase change	High-frequency micro vibration creates intermittent contact and the material softening effect	Using magnetic fields to regulate dislocation motion within the material	Implanting ions form an defect layer that weakens bonds in a shallow surface region
Cutting force	Large reduction	Moderate reduction	Reduction depending on the carrier material	Small reduction
Material removal rate	Enables increased depth of cut and feed rate	Enables steady removal but typically slower than LAM	Slight improvement	Slightly increase the depth of cut
Surface roughness	Significant improvement	Significant improvement	Significant improvement	Significant improvement
Subsurface damage	Significant reduction of brittle cracking	Micro-cracks, spalling, and pitting	Moderate reduction	Deep cracks and excessive amorphization
Cost	Medium	Medium	Medium	High
Typical side effects	HAZ, oxidation, and recast	Fixture fatigue and secondary ploughing	Field nonuniformity	Residual stress and foreign species

## Laser-assisted diamond turning of SiC

Diamond turning is widely used to achieve ultra-precision surfaces. However, its application to SiC remains challenging due to its pronounced tendency to undergo brittle fracture during machining. Although extensive research has enabled ductile mode machining of this type of material through brittle-to-ductile transition mechanisms, key limitations including low material removal rates and severe tool wear remain unresolved. To address these problems, laser-assisted diamond turning (LADT) has emerged as a promising approach. It can enhance the machinability of SiC and improve machining efficiency through thermal effects and localized surface modification.

**Fig. 11:**
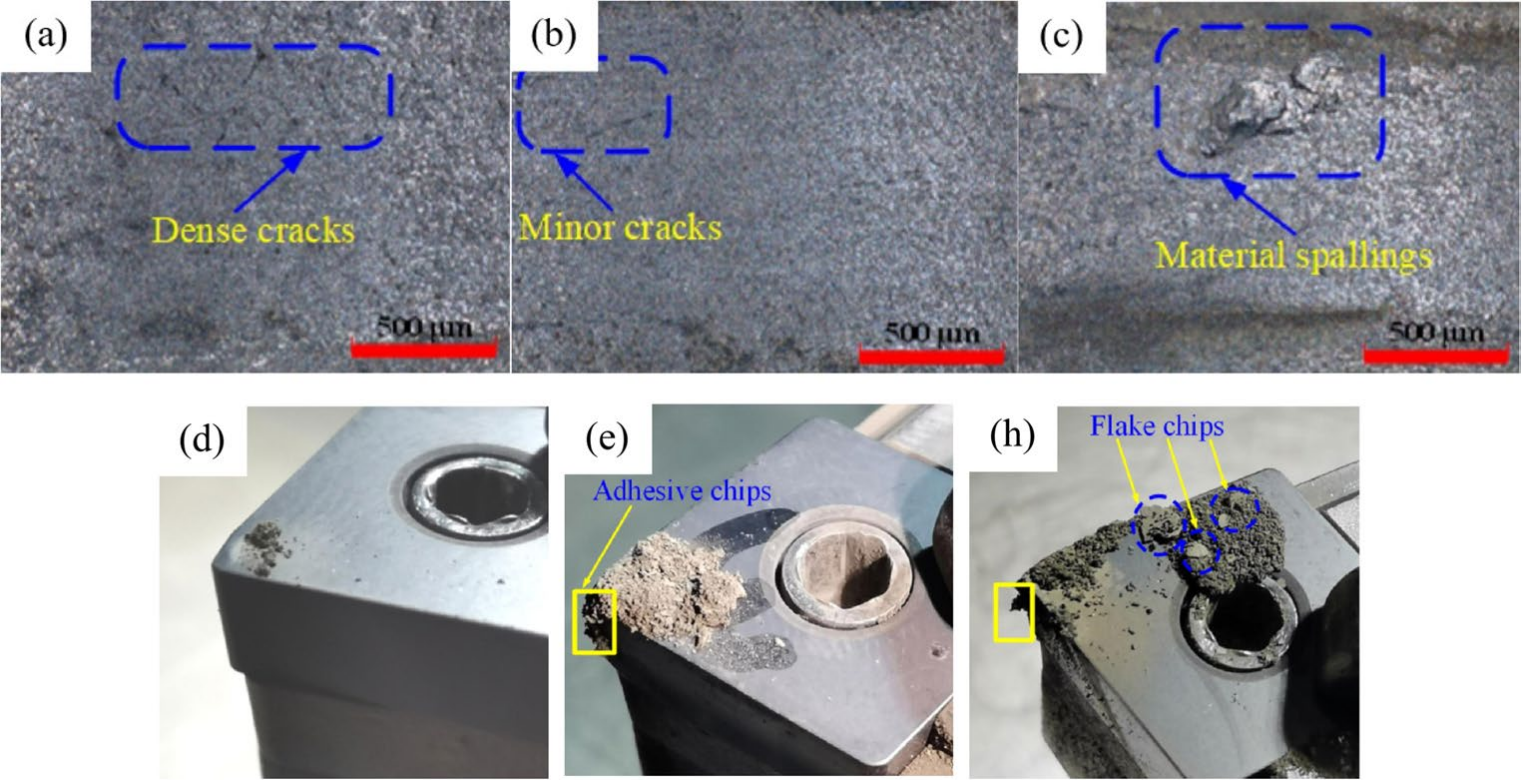
Machined surface morphology and chip adhesion on the diamond tool cutting edge at different laser power settings [77]. (**a**) Brittle-mode surface (**b**) Plastic-mode surface (**c**) Thermally damaged surface. (**d**) Chip adhesion after brittle-mode machining; (**e**) Chip adhesion after plastic-mode machining; (**f**) Chip adhesion after thermally damaged machining

In recent years, considerable efforts have been devoted to investigating a wide range of aspects related to laser-assisted turning for SiC. These studies have focused on the effects of laser heating on material removal modes, the formation of surface and subsurface damage, brittle-to-ductile transition behaviour, mechanisms of crack propagation, and the sensitivity of laser parameters on tool life. Cao [77] performed laser-assisted turning of pressureless-sintered SiC ceramics using a continuous-wave fibre laser and employed a Box–Behnken response surface methodology to develop regression models for predicting surface roughness and the maximum flank wear width. The results indicate that laser preheating facilitates the onset of the brittle–ductile transition during machining. As shown in Fig. 11, chip morphology progressed from granular debris in the brittle regime to micro-arc-shaped chips and continuously adherent chips in the plastic-cutting regime, and ultimately to large flake-like chips under thermal damage conditions. Surface cracks were no longer observed within the stable plastic-cutting window. Both Ra and VBmax decreased and then increased with rising laser power. The minimum Ra of 0.513 μm and the minimum VBmax of about 698.5 μm were obtained at a laser power of approximately 225 W. Sensitivity analysis further revealed that the importance ranking of factors for Ra followed the order: cutting depth, spindle speed, feed rate, laser power. In contrast, for VBmax, the factor significance ranked as: cutting depth, laser power, spindle speed, feed rate.

**Fig. 12:**
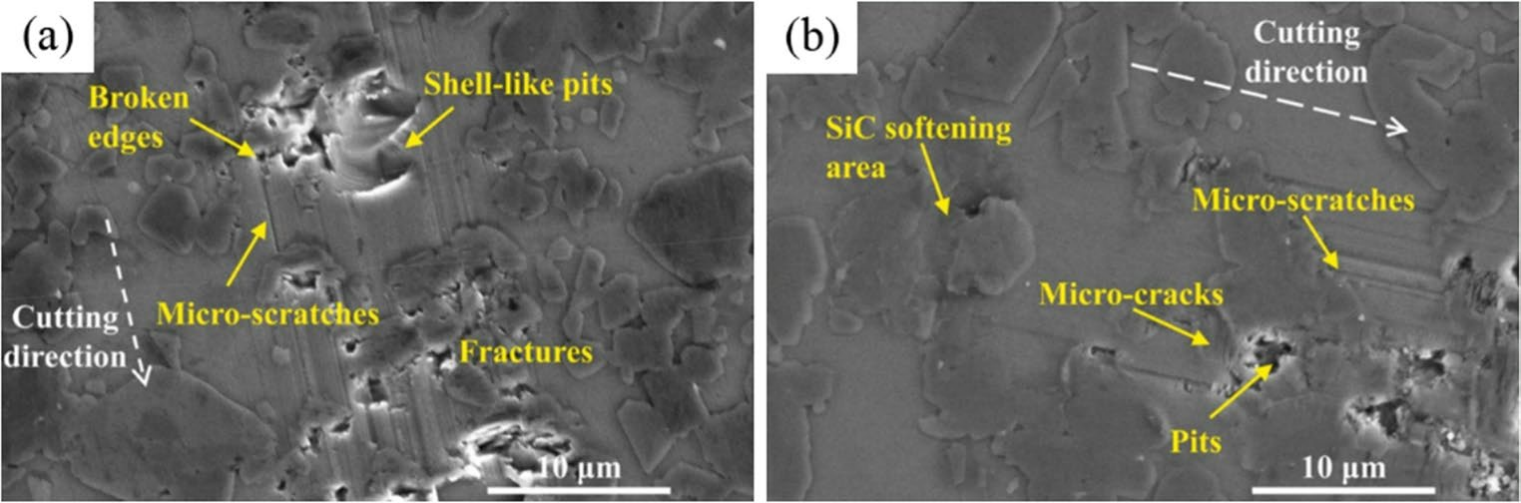
Nano-scratch test micro-groove morphology [79]. (**a**) Machining without laser assistance (**b**) Machining with laser assistance

Dai [78] established a thermo-mechanical coupled finite element model for SiC based on the Johnson–Holmquist 2 constitutive law, in which a pre-set temperature field was used to emulate laser preheating. In parallel, an experimental platform was constructed, combining a cubic boron nitride tool with a continuous-wave laser. The results show that high-temperature softening promotes a transition in SiC removal from brittle fracture to predominantly plastic and near-plastic deformation regimes. The chip morphology correspondingly changes from powdery or fragmented debris to continuous ribbon-like chips. Cracks and edge chipping are markedly reduced under laser-assisted conditions. The surface roughness decreases from over 1 μm to about 0.5–0.8 μm, and tool wear was accordingly reduced. The relative influence on cutting behaviour was ranked as follows: feed rate, depth of cut, laser power, and spindle speed, with the interaction between depth of cut and feed rate exhibiting the strongest effect. Zhang [79] investigated laser-assisted diamond turning of RB-SiC using a continuous-wave laser. The results indicated that high temperature generated by the laser reduced stress concentration, lattice defects, the risk of surface damage and crack propagation, thereby significantly increasing the critical depth for brittle-to-ductile transition. This thermal effect enhanced the plastic deformation capability of RB-SiC. As shown in Fig. 12, during machining without laser assistance, the Si phase maintained stable ductile cutting, whereas the SiC phase experienced grain pull-out or edge cracking. With LAM, the SiC phase softened, reducing the brittle-to-ductile transition differences between the Si and SiC phases, leading to more uniform cutting behaviour between these two phases. After softening, the SiC phase tended to undergo transgranular fracture rather than intergranular fracture. This reduced micro-cracks and surface damage, thereby sustaining ductile mode cutting.

**Fig. 13:**
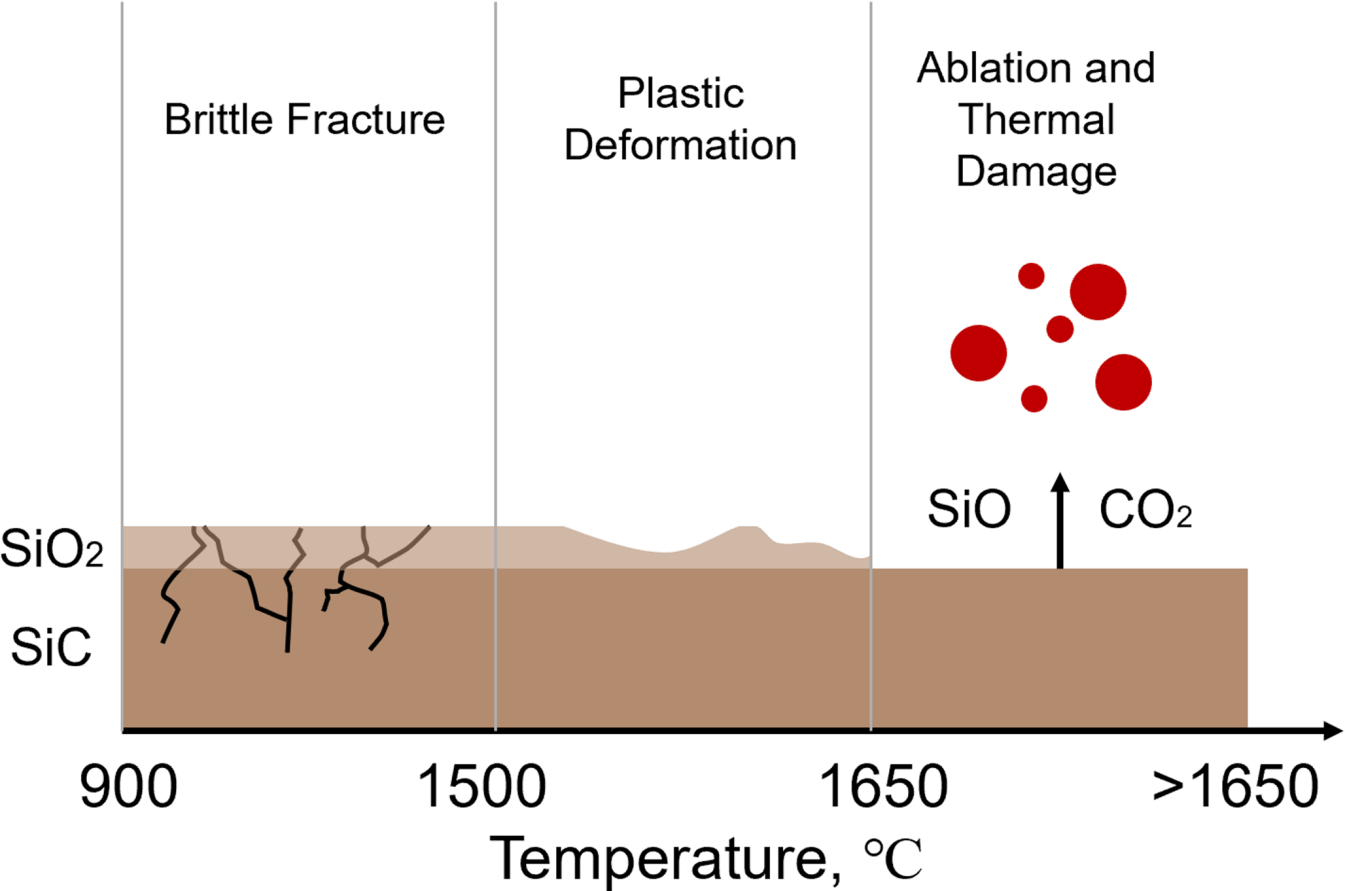
Thermal effects on mechanisms in laser-assisted turning

Dai [80] conducted laser-assisted turning experiments on SiC using a continuous-wave laser and employed both the orthogonal method and response surface methodology (RSM) for machining parameters optimization. The study focused on optimizing laser power, cutting depth, rotational speed, and feed rate to enhance surface quality while minimizing surface roughness. The results showed that laser power and cutting depth were the most significant factors affecting surface roughness, followed by rotational speed, whereas feed rate was identified as the least influential factor. Cao [81] developed a 3D transient heat transfer model and conducted laser-assisted turning experiments on SiC using a continuous-wave laser to validate the model predictions. As shown in Fig. 13 the study found that a SiO₂ protective layer formed on the SiC surface when the temperature was between 900 °C and 1650 °C, inhibiting further oxidation diffusion. However, once the temperature exceeded approximately 1650 °C, the protective layer deteriorated, leading to the formation of gaseous SiO and CO₂. These reactions accelerated surface ablation and caused substantial material loss. When the SiC temperature was below 1500 °C, the yield strength exceeded the fracture strength, and material removal was dominated by brittle fracture. In the intermediate temperature range between 1500 °C and 1650 °C, the material softened sufficiently for removal to occur predominantly through plastic deformation. When the temperature exceeded 1650 °C, material removal transitioned to ablation-dominated mechanisms driven by severe thermal damage.

The above studies have demonstrated that LAM reduces tool wear and enhances machining efficiency. However, most existing research primarily employs continuous-wave lasers for laser assisted machining. The continuous heating characteristics of continuous-wave lasers often lead to an expanded heat-affected zone during machining. This expanded heat-affected zone induces thermal expansion and phase transformation in SiC, leading to surface cracks, microscopic defects, and thermal damage, collectively degrading machining accuracy and surface integrity. In contrast, pulse lasers enable high-precision surface modification within highly localized regions owing to their short pulse widths and high peak power, thereby reducing the heat-affected zone and improving machining accuracy and surface quality. However, research on pulse laser assisted turning of SiC comprehensive understanding is still lacking, particularly regarding the control of thermal effects, the understanding of material removal mechanisms, and the maintenance of surface integrity during machining.

## Advances in SiC surface modification with ultrashort lasers

### Characteristics and advantages of pulse laser

Pulse lasers utilize short-duration, high-energy pulses to achieve instantaneous localized heating, thereby effectively minimizing the heat-affected zone and reducing the associated thermal stress and thermal damage. This transient thermal effect helps control the surface modification process, concentrating modifications within a specific region while preserving the structural stability of adjacent regions. Additionally, with their high peak power and precise energy control capabilities, pulse lasers can be tailored to the thermophysical behaviour of diverse materials, making them particularly suitable for machining high-hardness, brittle, and thermally sensitive materials. Consequently, pulse lasers have garnered substantial research interest in recent years and have emerged as a promising tool for ultra-precision machining. During laser-assisted machining, the removed material primarily originates from the heat-affected zone of the workpiece surface by laser irradiation [83]. Researchers believe that using pulse lasers reduces the interaction time between the laser and the material, thereby limiting heat diffusion and improving machining precision.

**Fig. 14:**
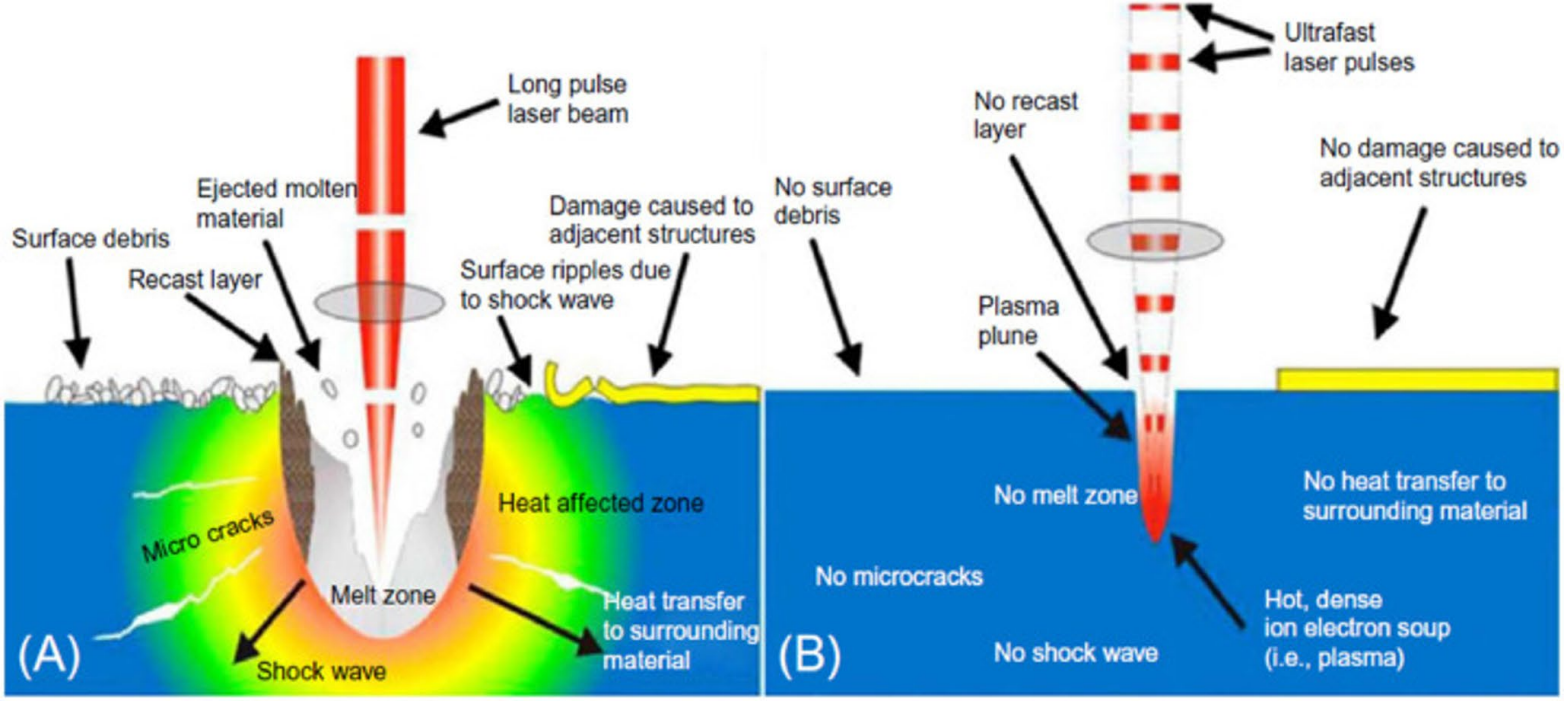
Thermal interaction with the target material. (**a**) Long-pulse lasers (**b**) Short-pulse lasers [82]

Pulse lasers can be classified according to their wavelength into long-pulse lasers (millisecond lasers and microsecond lasers), short-pulse lasers (nanosecond lasers), and ultrashort pulsed lasers (picosecond lasers and femtosecond lasers). Both long-pulse and short-pulse lasers utilize localized heating effects to soften materials, thereby reducing cutting forces. As shown in Fig. 14, due to the longer pulse duration, long-pulse lasers tend to generate more significant thermal damage and larger heat-affected zones than short-pulsed lasers, making them more prone to generating residual stress and micro-cracks. In contrast, with smaller heat-affected zones, short-pulse lasers are more effective at preserving material structure and controlling the modification area.

**Fig. 15:**
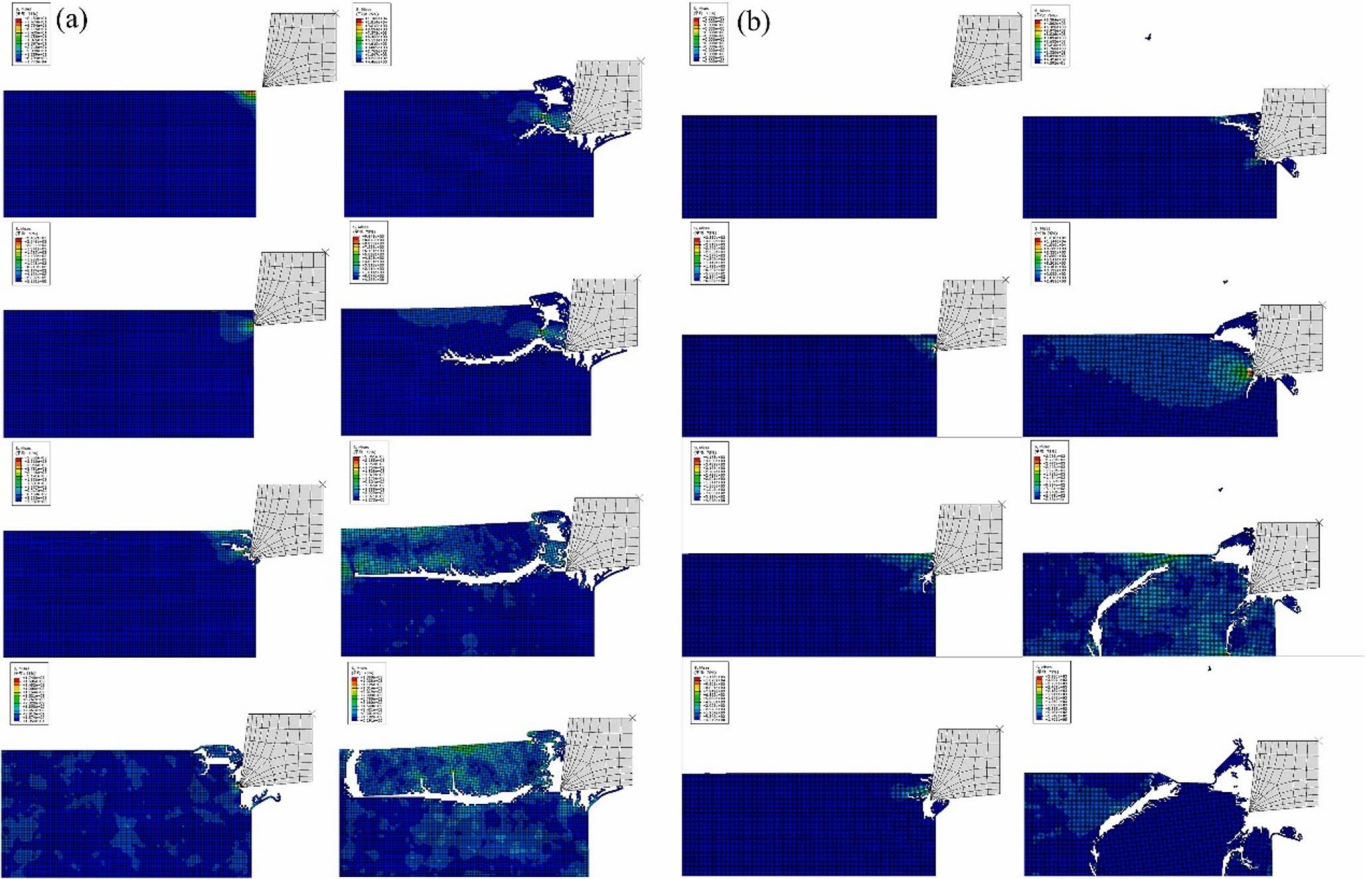
Turning simulation of Zerodur (**a**) With pulsed laser assistance (**b**) Without pulsed laser assistance [85]

Tadavani [83] conducted turning experiments on Inconel 718 superalloy using a long-pulsed laser. The study found that by optimizing parameters such as pulse duration, frequency, average power, and beam diameter, both the surface temperature and depth of the heat-affected zone could be effectively controlled. The surface elemental distribution after pulse laser-assisted turning and conventional machining was uniform, without observable microstructural differences. Pulse laser processing did not alter the intrinsic material properties of the alloy. With pulse laser assistance, the specific cutting energy was significantly reduced, thereby enhancing the machinability and noticeably reducing tool wear. Liu [84] investigated the effect of laser parameters on tool wear during turning of Al-50wt%Si alloy using a short-pulsed laser. The results showed that with increasing laser power, the dominant tool wear mechanism shifted from abrasive wear to adhesive wear and subsequently to oxidative wear. A higher laser power significantly reduced the overall extent of tool wear. However, an excessive increase in laser power can lead to elevated high temperatures, causing chip adhesion and promoting severe adhesive and oxidative wear, thereby accelerating tool wear. As the pulse width increases, the duration of thermal effects also extends, leading to more severe thermal damage and material deformation. It can cause micro-cracks and edge chipping on the tool. Increasing the pulse frequency shortens the cooling time between pulses reduced temperature fluctuations, resulting in smoother and more uniform temperature rises, resulting in progressively reducing tool wear. When the laser pulse frequency reached 400 kHz, the temperature of cutting zone stabilized, and further increases in frequency no longer contributed to additional thermal accumulation. Guo [85] conducted both numerical simulations and machining experiments on Zerodur using nanosecond pulsed laser-assisted synchronous servo turning. The results showed that under conventional machining conditions, increasing the cutting depth during tool movement increased the likelihood of longitudinal crack formation in the workpiece. As shown in Fig. 15, the application of pulsed lasers reduced the occurrence of longitudinal cracks but may increase the tendency of transverse crack formation. Higher laser power caused heat accumulation and produced a more uniform surface temperature distribution across the sample, thereby reducing shear stress, cutting forces, and surface damage.

Preheating and softening the material ahead of the tool, thereby reducing its effective strength, constitute one of the widely adopted principles of LAM. This approach has been extensively employed in machining processes assisted by continuous-wave, long-pulsed, and short-pulsed lasers. Although this method improves machinability by promoting thermal softening, precisely controlling the heat-affected zone and avoiding unintended microstructural alterations remain significant challenges.

**Fig. 16:**
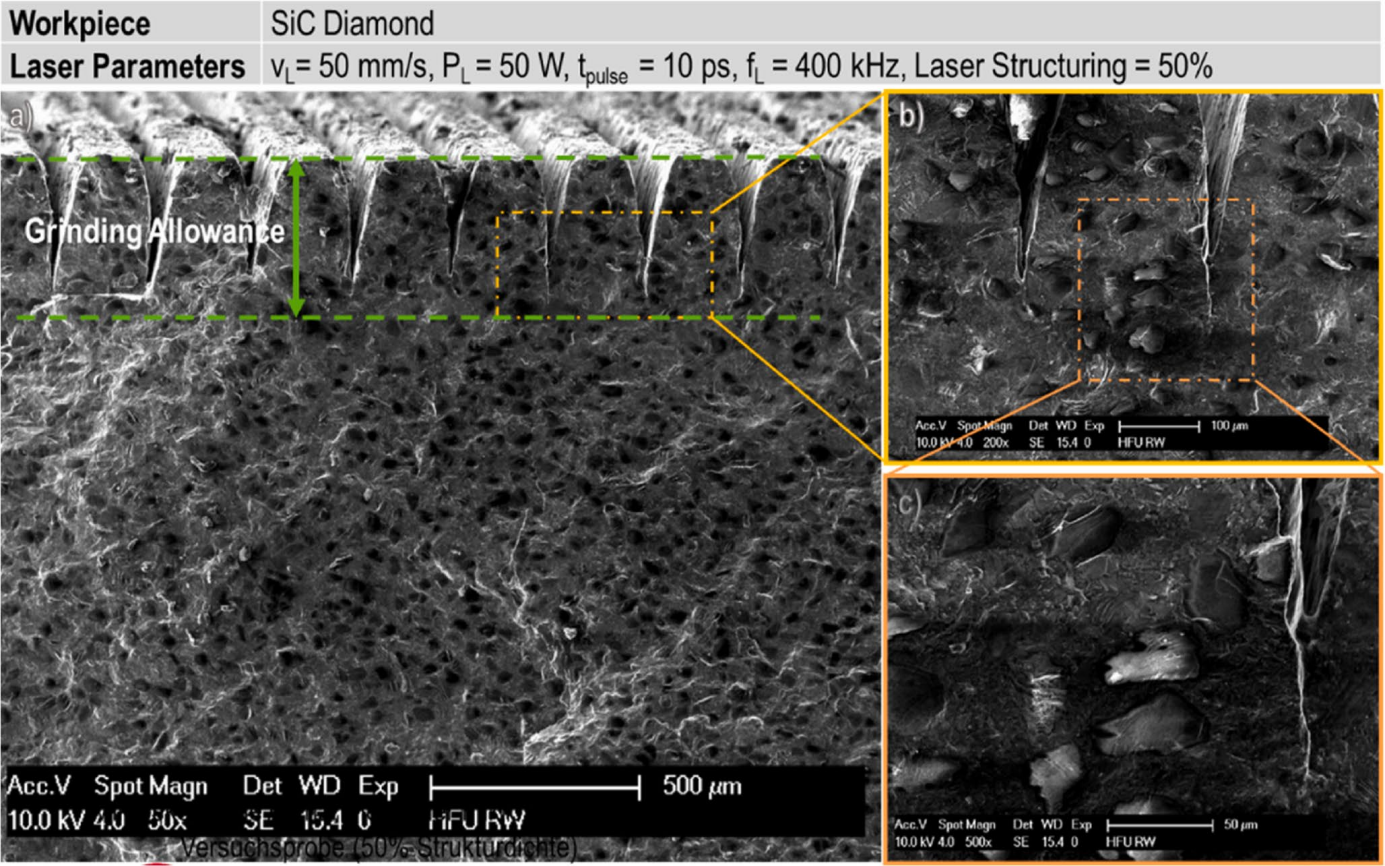
Laser-structured the surface of SiC-bonded diamond materials [86]

With the continuous advancements of laser technology, featuring extremely high peak power and ultra-short pulse durations, ultra-short pulse lasers have addressed key limitations of traditional pulsed lasers. Compared to long-pulsed and short-pulsed lasers, ultra-short pulse lasers deposit energy into the material surface on picosecond or femtosecond timescales, which are significantly shorter than the characteristic thermal diffusion times. This non-thermal processing capability allows material modification to occur before heat conduction takes place, significantly reducing thermal diffusion effects and enabling higher machining precision. The resulting a thermal material-removal behaviour provides an alternative approach for laser-assisted machining. Furthermore, by structuring the workpiece surface and forming a modified layer, ultrashort-pulse irradiation can reduce cutting forces, ease machining resistance and enhance the overall machinability of HBMs. Paknejad [86] employed an ultra-short pulse laser to form microstructures, as shown in Fig. 16, together with controllable micro-cracks and subsurface damage on the surface of SiC-bonded diamond materials. The laser-modified specimens were subsequently subjected to grinding. The results demonstrated that laser structuring increased the depth of cut per pass, reduced cutting forces, and significantly enhanced machining efficiency. During grinding, these microstructures reduced the material volume requiring removal. Moreover, the controllable micro-cracks and subsurface damage generated by the laser reduced the local material strength. When the overlap rate reached 170%, the grinding process removed only the laser-induced micro-cracks and subsurface damage regions. High-overlap laser treatment generates denser micro-cracks and grooves, which enhance coolant penetration, reduce tool load, and minimize heat accumulation. Performing mechanical machining after high-overlap laser modification using ultra-short lasers is significantly more efficient than machining the material in its unmodified state.

**Fig. 17:**
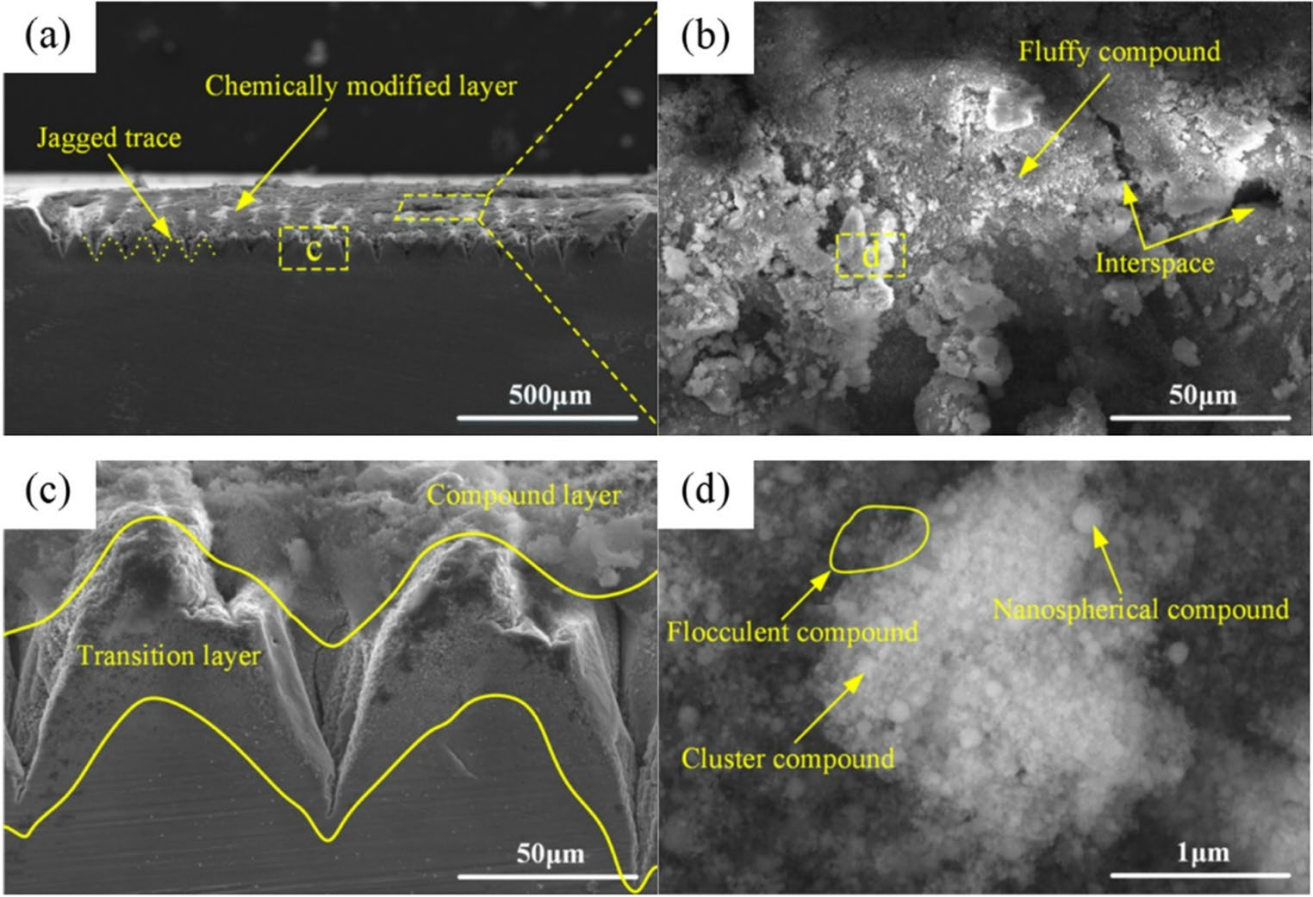
(**a**) Surface modification area of Ti6Al4V. (**b**) surface of modification area. (**c**) cross section of modified layer. (**d**) clusters of nanosized particles [87]

Xiao [87] employed a picosecond laser to induce surface modification in Ti6Al4V, after which the modified layer was subsequently removed through a grinding process. The results showed that the picosecond laser generated a modified layer on Ti6Al4V. As shown in Fig. 17, the modified layer consists of a compound layer and an underlying transition layer. The compound layer consists of clusters of nanoscale particles, including TiN, TiO₂, and Al₂O₃, exhibiting a loose and easily detachable morphology. The transition layer, characterized by grain precipitation and microcracks formation, promotes plastic deformation and improves the material removal efficiency of Ti6Al4V. The formation mechanism of this structure involves laser-induced lattice vibration enhancement and rapid temperature rise, which trigger oxidation and nitridation reactions. These processes lead to surface element redistribution and chemical bond reconstruction, resulting in the formation of new phases and microcracks. Compared to conventional grinding, the laser modified surface markedly reduced grinding force, while the surface roughness decreased by 62.5%.

Recent studies have demonstrated that the non-thermal ablation mechanism characteristic of ultra-short pulse lasers can significantly reduce the heat-affected zone, the formation of micro-cracks and surface damage, while simultaneously enabling higher precision in surface modification. Surface modification and structuring induced by ultra-short pulse lasers can significantly enhance the machinability of HBMs, thereby reducing tool wear, improving surface quality and increasing machining accuracy.

### Surface modification of SiC via ultrashort pulsed lasers

Ultra-short pulse laser technology enables precise energy delivery on picosecond or even femtosecond timescales, providing exceptionally high machining flexibility and precision of control. This capability renders it particularly suitable for the precision machining of HBMs, including SiC. However, the laser machining response of SiC under laser irradiation is influenced by a range of interacting process parameters. As summarized in Table 3, these variables govern the underlying interaction mechanisms between the laser beam and SiC. These interactions fall predominantly into two categories, thermal and photochemical mechanisms. Thermal mechanisms include heating, melting, and vaporization, whereas photochemical mechanisms involve bond formation and bond breaking.

**Table 3:** Factors influencing laser machining performance [88]

Variable type	Variable name
Laser parameters	Power
	Temporal mode
	Spatial mode
	Wavelength
	Raw beam diameter
	Polarization
	Depth of focus
	Projected spot size
	Focal plane position
Material properties	Composition
	Absorptivity
	Thermal conductivity
	Specific heat capacity
	Latent heats
	Thermal expansion coefficient
	Transformation temperatures
	Thickness
Processing conditions	Traverse rate
	Process gases
	Initial temperature

**Fig. 18:**
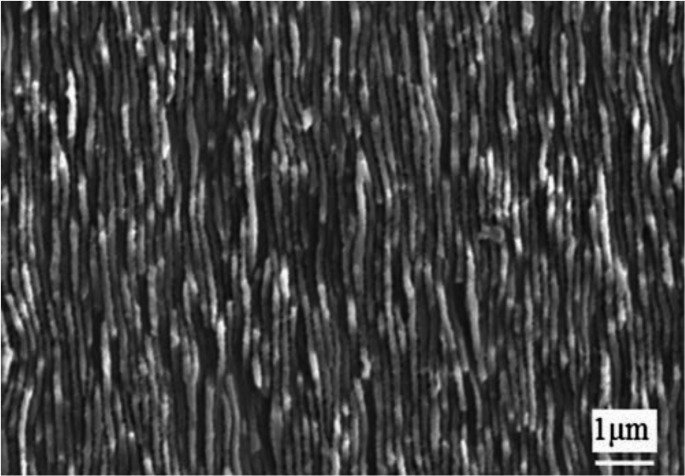
Periodic structures formed on the surface of SiC by ultra-short laser irradiation [89]

For SiC applications, although research on ultra-short lasers for assisted mechanical machining remains relatively limited, several studies on laser induced surface modification of SiC have been reported. Wang [89] performed femtosecond laser modification on 4 H-SiC, followed by CMP. As shown in Fig. 18, the study found that the femtosecond laser induced periodic surface structures approximately 150 nm in size,, together with an oxidation induced amorphous layer. These periodic structures increased the contact area with the polishing slurry, thereby enhancing the efficiency of chemical reactions and improving the material removal rate. Additionally, the formation of the SiO₂ amorphous layer reduced the surface hardness of SiC, further improving machining efficiency and the resulting surface quality.

Gao [90] performed picosecond laser irradiation on 6 H-SiC to investigate the associated surface modification mechanisms. The study found that under picosecond laser irradiation, the strong C-Si bonds were oxidized to form less-stable oxygen-containing bonds, including C-O bonds, Si-C-O bonds, and Si-O bonds. Consequently, these bond conversions resulted in the formation of polycrystalline, oxygen-rich surface layer on the laser irradiated region. Liu [91] systematically investigated picosecond laser induced surface modification of 4 H-SiC. The results showed that the surface roughness increased initially with rising energy density and subsequently decreased. Additionally, the laser induced periodic ripple structures progressively diminished as the energy density increased. The increase in surface roughness was attributed to phase explosion and thermal effects at a high energy density condition, which enhanced particle redeposition on the SiC surface. As the energy density further increased, plasma absorbed laser energy through inverse bremsstrahlung radiation, leading to the formation of a plasma shielding effect. This shielding redistributed the absorbed energy more uniformly within the laser irradiated zone, thereby reducing the resulting surface roughness. The formation of microstructures occurred at relatively low energy densities, where the interaction between the laser and the material was dominated by slight melting and evaporation. These periodic microstructures were caused by electromagnetic wave interference and surface plasmon wave coupling during laser irradiation. As the energy density increased further, the interaction gradually transitions to phase explosion, causing the periodic microstructures to disappear. Under laser irradiation, significant oxidation occurs on the surface and subsurface of SiC, generating a large amount of SiO₂ and resulting in a notable decrease in hardness. This process facilitates subsequent mechanical machining.

Previous studies have shown that ultra-short pulse laser surface modification can effectively enhance the machinability of SiC. However, research on ultra-short pulse laser assisted diamond turning is still in its early stage, and substantial knowledge gaps remain, particularly regarding the laser induced surface modification mechanisms and the machining response mechanisms relevant to diamond turning. SiC possesses excellent properties, making it suitable for various applications. However, its inherently poor machinability remains a major barrier to its wider industrial adoption.

As discussed above, achieving low cost, high efficiency, and nanometre-level precision simultaneously in freeform SiC machining continues to pose a major challenge. Ultrashort-pulse lasers, through the formation of laser-induced structured surfaces and targeted surface modifications, provide a promising solution. These surface features not only promote material softening and chip formation but also enhance tool-material interaction and reduce subsurface damage. More importantly, the ultrashort pulse duration offers the potential to overcome the inherent limitations of traditional laser-assisted machining, particularly in controlling the extent of material modification caused by enlarged heat-affected zones. As laser–material interaction mechanisms become highly tuneable via ultrafast pulse control, this technique is expected to greatly change the current approach to precision SiC machining. Ultimately, the integration of laser-induced structuring with ultraprecision turning is expected to drive the evolution from conventional cutting toward ultrashort-pulse laser assisted approaches, paving the way for a new generation of scalable, precise, and intelligent machining technologies.

## Conclusion

This review has presented a comprehensive overview of the underlying mechanisms and recent advances in laser-assisted cutting and laser-induced surface modification of SiC. Beginning with the inherent limitations of conventional SPDT, the review further surveyed arrange of field-assisted techniques developed to enhance the machinability of HBMs. Among these techniques, laser-assisted machining has demonstrated significant potential in reducing cutting forces, prolonging tool life, and improving both surface integrity and subsurface damage suppression.

- Laser assistance expands the plastic-dominated material-removal window, stabilizes chip formation behaviour, and enhances both surface integrity and tool life relative to conventional mechanical cutting.
- By elevating the near-surface region temperature into a controlled plastic-deformation regime, LAM facilitates the brittle-to-ductile transition, stabilizes chip formation, and suppresses micro-cracking and edge chipping. The consequent reductions in cutting force and tool–chip contact stress yield lower flank wear and extended tool life, while surface integrity is enhanced through diminished subsurface damage and improved surface roughness.
- In pulsed laser regimes and ultrashort-pulsed laser regimes, the highly localized energy deposition further limits the extent of heat affected zone, enables targeted surface modification, and creates opportunities to integrate pre-conditioning, turning, and light finishing into a compact machining process.

By integrating ultrashort pulsed laser structuring with ultraprecision diamond turning, a viable hybrid machining pathway can be established that overcomes the long-standing bottleneck of simultaneously achieving low cost, high efficiency, and nanoscale precision in the fabrication of freeform SiC surfaces. However, the combination of ultrashort pulsed lasers and single-point diamond turning for SiC machining still faces substantial scientific and technological challenges.

- The challenge of achieving higher material removal rates while simultaneously maintaining nanoscale surface quality and form accuracy has not yet been fully resolved.
- Although pulsed and ultrashort-pulse irradiation can confine energy deposition and reduce the heat affected zone, achieving reliable control of thermal effects and maintaining surface integrity during turning remain challenging. Heat accumulation, crack-mode transitions, and temperature-driven defect formation collectively hinder scaling the process to higher material-removal rates.
- Research that directly connects ultrashort-pulse-induced phase transformations, chemical modifications and microcracks reorientation and density evolution with the corresponding reductions in cutting force, tool wear, and subsurface damage observed during the subsequent turning stage remains notably limited.

Future research should focus on optimizing laser parameters to achieve selective and spatially confined energy deposition, as well as refining surface structuring strategies to better control modification depth, reduce defect formation, and enhance overall process precision. Ultimately, integrating ultrafast laser technologies into SiC machining workflows is anticipated to enable industrial-scale, nanometric-precise, and cost-effective fabrication of complex freeform surfaces.

## Data Availability

Not applicable.

## References

[1] Li J, Yang G, Liu X, Luo H, Xu L, Zhang Y, Cui C, Pi X, Yang D, Wang R (2022) Dislocations in 4H silicon carbide. J Phys D Appl Phys 55:463001. 10.1088/1361-6463/ac8a58

[2] La Via F, Alquier D, Giannazzo F, Kimoto T, Neudeck P, Ou H, Roncaglia A, Saddow SE, Tudisco S (2023) Emerging SiC applications beyond power electronic devices. Micromachines 14:1200. 10.3390/mi14061200 37374785 10.3390/mi14061200 PMC10300968

[3] Zhang X, Hu H, Wang X, Luo X, Zhang G, Zhao W, Wang X, Liu Z, Xiong L, Qi E, Cui C, Wang Y, Li Y, Wang X, Li L, Bai Y, Cheng Q, Zhang Z, Li R, Tang W, Zeng X, Deng W, Zhang F (2022) Challenges and strategies in high-accuracy manufacturing of the world’s largest SiC aspheric mirror. Light Sci Appl 11:310. 10.1038/s41377-022-00994-3 36284086 10.1038/s41377-022-00994-3 PMC9596426

[4] Yao X, Zhou T, Yu Q, He Y, Su X, Zhao B, Wang X, Zhang Z (2024) Template-oriented-assembly microsphere lithography for multi-type SiC microlens arrays. Appl Surf Sci 673:160857. 10.1016/j.apsusc.2024.160857

[5] Zheng S, Zhang B, Liu X, Chen Z, Huang Z, Yin J (2023) β→ α phase transformation and properties of solid-state-sintered SiC ceramics with tac addition. Materials 16:3787. 10.3390/ma16103787 37241413 10.3390/ma16103787 PMC10223872

[6] Du W, Ma B, Thomas J, Singh D (2023) Concurrent reaction-bonded joining and densification of additively manufactured silicon carbide by liquid silicon infiltration. J Eur Ceram Soc 43:2345–2353. 10.1016/j.jeurceramsoc.2023.01.032

[7] Borrero-López O, Ortiz AL, Guiberteau F, Padture NP (2007) Sliding-wear‐resistant liquid‐phase‐sintered SiC processed using α‐SiC starting powders. J Am Ceram Soc 90:541–545. 10.1111/j.1551-2916.2006.01421.x

[8] Xu TT, Cheng S, Jin LZ, Zhang K, Zeng T (2020) High-temperature flexural strength of SiC ceramics prepared by additive manufacturing. Int J Appl Ceram Technol 17:438–448. 10.1111/ijac.13454

[9] Wang XL, Gao XD, Zhang ZH, Chen LS, Ma HP, Yang WM (2021) Advances in modifications and high-temperature applications of silicon carbide ceramic matrix composites in aerospace: a focused review. J Eur Ceram Soc 41:40671–44688. 10.1016/j.jeurceramsoc.2021.03.051

[10] Li Z, Xiao P, Zhang BG, Li Y, Lu YH, Zhu SH (2016) Preparation and dynamometer tests of 3D needle-punched C/C-SiC composites for high-speed and heavy-duty brake systems. Int J Appl Ceram Technol 13:423–433. 10.1111/ijac.12514

[11] Qiu H, Yu J, Zheng S, Yao Y, Song P, Chen H, Wu Y (2024) Effect of heat transfer and storage ability of silicon carbide (SiC) ceramic particles on the microwave deicing characteristics of cement-based materials. Ceram Int 50:17848–17860. 10.1016/j.ceramint.2024.02.273

[12] Steen M, Ranzani L (2000) Potential of SiC as a heat exchanger material in combined cycle plant. Ceram Int 26:849–854. 10.1016/S0272-8842(00)00027-4

[13] Latypova L, Murzakhanov F, Mamin G, Sadovnikova M, Von Bardeleben HJ, Rau JV, Marat G (2024) Exploring high-spin color centers in wide band gap semiconductors SiC: a comprehensive magnetic resonance investigation (EPR and ENDOR analysis). Molecules 29:3033. 10.3390/molecules29133033 38998983 10.3390/molecules29133033 PMC11243473

[14] Kleppinger JW, Chaudhuri SK, Karadavut O, Nag R, Watson DLP, McGregor DC, Mandal KC (2022) Deep-level transient spectroscopy and radiation detection performance studies on neutron irradiated 250-µm-thick 4H-SiC epitaxial layers. IEEE Trans Nucl Sci 69:1972–1978. 10.1109/TNS.2022.3168789

[15] Tao Z, Li YT, Chen Y, Feng XY, Zhu XY, Chen ZX, Yao J, Zheng YC, Cai JC, Song HQ, Sun SY (2021) Review on space energy. Appl Energy 292:116896. 10.1016/j.apenergy.2021.116896

[16] Rogalski A, Chrzanowski K (2017) Infrared devices and techniques. In: Dakin JP, Brown RGW (eds) Handbook of Optoelectronics. CRC Press, Boca Raton, pp 633–686. 10.1201/9781315157009-18

[17] Akbar G, Di Fatta A, Rizzo G, Ala G, Romano P, Imburgia A (2024) A detailed review of partial discharge detection methods for SiC power modules under square-wave voltage excitation. Energies 17:5793. 10.3390/en17225793

[18] Haynes WM (2016) CRC Handbook of Chemistry and Physics. CRC Press, Boca Raton, pp 55–120. 10.1201/9781315380476

[19] Casady JB, Johnson RW (1996) Status of silicon carbide (SiC) as a wide-bandgap semiconductor for high-temperature applications: A review. Solid State Electron 39:1409–1422. 10.1016/0038-1101(96)00045-7

[20] Han R, Xu X, Hu X, Yu N, Wang J, Tian Y, Huang W (2003) Development of bulk SiC single crystal grown by physical vapor transport method. Opt Mater 23:415–420. 10.1016/S0925-3467(02)00330-0

[21] Rao X, Zhang F, Lu Y, Luo X, Chen F (2020) Surface and subsurface damage of reaction-bonded silicon carbide induced by electrical discharge diamond grinding. Int J Mach Tools Manuf 154:103564. 10.1016/j.ijmachtools.2020.103564

[22] Agarwal S, Rao PV (2008) Experimental investigation of surface/subsurface damage formation and material removal mechanisms in SiC grinding. Int J Mach Tools Manuf 48:698–710. 10.1016/j.ijmachtools.2007.10.013

[23] Ke J, Fu Y, Liu C, Zhang JG, Chen X, Xu JF (2024) Investigation on system design methodology and cutting force optimization in laser-assisted diamond machining of single-crystal silicon. J Manuf Process 115:1–17. 10.1016/j.jmapro.2024.01.081

[24] Wang W, Lu X, Wu X, Zhang Y, Wang R, Yang D, Pi X (2023) Chemical–mechanical polishing of 4H silicon carbide wafers. Adv Mater Interfaces 10:2202369. 10.1002/admi.202202369

[25] Wang W, Lu X, Wu X, Wang R, Yang D, Pi X (2025) Oxidation anisotropy of 4H-SiC wafers during chemical-mechanical polishing. Mater Sci Semicond Process 185:109014. 10.1016/j.mssp.2024.109014

[26] Wu M, Huang H, Wu Y, Xu Z, Li T, Macleod I, Wu X (2024) Mechanism of friction-induced chemical reaction high-efficient polishing single crystal 4H-SiC wafer using pure iron. Tribol Int 193:109450. 10.1016/j.triboint.2024.109450

[27] Zong WJ, Li ZQ, Sun T, Cheng K, Li D, Dong S (2010) The basic issues in design and fabrication of diamond-cutting tools for ultra-precision and nanometric machining. Int J Mach Tools Manuf 50:411–419. 10.1016/j.ijmachtools.2009.10.015

[28] Patten J, Gao W, Yasuto K (2005) Ductile regime nanomachining of single-crystal silicon carbide. J Manuf Sci Eng 127:522–532. 10.1115/1.1949614

[29] Patten JA, Jacob J (2008) Comparison between numerical simulations and experiments for single-point diamond turning of single-crystal silicon carbide. J Manuf Process 10:28–33. 10.1016/j.jmapro.2008.08.001

[30] Ajjarapu SK, Patten JA, Cherukuri H, Brand C (2004) Numerical simulations of ductile regime machining of silicon nitride using the Drucker-Prager material model. Proc Institution Mech Eng Part C: J Mech Eng Sci 218:577–582. 10.1243/095440604774202204

[31] Yan J, Zhang Z, Kuriyagawa T (2009) Mechanism for material removal in diamond turning of reaction-bonded silicon carbide. Int J Mach Tools Manuf 49:366–374. 10.1016/j.ijmachtools.2008.12.007

[32] Goel S, Luo X, Comley P, Reuben RL, Cox A (2013) Brittle–ductile transition during diamond turning of single crystal silicon carbide. Int J Mach Tools Manuf 65:15–21. 10.1016/j.ijmachtools.2012.09.001

[33] Blake PN, Scattergood RO (1990) Ductile-regime machining of germanium and silicon. J Am Ceram Soc 73:949–957. 10.1111/j.1151-2916.1990.tb05142.x

[34] Huang W, Yan J (2023) Mechanisms of tool-workpiece interaction in ultraprecision diamond turning of single-crystal SiC for curved microstructures. Int J Mach Tools Manuf 191:104063. 10.1016/j.ijmachtools.2023.104063

[35] Du PF, Chen WS, Deng J, Zhang SJ, Zhang JJ, Liu YX (2023) A critical review of piezoelectric ultrasonic transducers for ultrasonic-assisted precision machining. Ultrasonics 135:107145. 10.1016/j.ultras.2023.107145 37643548 10.1016/j.ultras.2023.107145

[36] Melkote S, Kumar M, Hashimoto F, Lahoti G (2009) Laser assisted micro-milling of hard-to-machine materials. CIRP Ann 58:45–48. 10.1016/j.cirp.2009.03.053

[37] To S, Wang H, Jelenković EV (2013) Enhancement of the machinability of silicon by hydrogen ion implantation for ultra-precision micro-cutting. Int J Mach Tools Manuf 74:50–55. 10.1016/j.ijmachtools.2013.07.005

[38] Yip WS, To S (2017) Reduction of material swelling and recovery of titanium alloys in diamond cutting by magnetic field assistance. J Alloy Compd 722:525–531. 10.1016/j.jallcom.2017.06.167

[39] Xing Y, Liu Y, Yin T, Li D, Sun Z, Xue C, Yip WS, To S (2024) Magnetic and ultrasonic vibration dual-field assisted ultra-precision diamond cutting of high-entropy alloys. Int J Mach Tools Manuf 202:104208. 10.1016/j.ijmachtools.2024.104208

[40] Yip WS, To S, Sun Z (2021) Hybrid ultrasonic vibration and magnetic field assisted diamond cutting of titanium alloys. J Manuf Process 62:743–752. 10.1016/j.jmapro.2020.12.037

[41] Azarhoushang B, Tawakoli T (2011) Development of a novel ultrasonic unit for grinding of ceramic matrix composites. Int J Adv Manuf Technol 57:945–955. 10.1007/s00170-011-3347-x

[42] Zheng Y, Wang C, Ma J, Li H, Li Y, Luo C (2023) Gradient characteristics of surface metamorphic layer microstructure induced by longitudinal torsional ultrasonic-assisted milling Ti-6Al-4V alloy. J Mater Eng Perform 32:10141–10157. 10.1007/s11665-023-07845-1

[43] Zhang C, Song Y (2019) A novel design method for 3D elliptical vibration-assisted cutting mechanism. Mech Mach Theory 134:308–322. 10.1016/j.mechmachtheory.2019.01.007

[44] Liu X, Wu D, Zhang J, Hu X, Cui P (2019) Analysis of surface texturing in radial ultrasonic vibration-assisted turning. J Mater Process Technol 267:186–195. 10.1016/j.jmatprotec.2018.12.021

[45] Kurniawan R, Ahmed F, Ali S, Park GC, Ko TJ (2021) Analytical, FEA, and experimental research of 2D-vibration assisted cutting (2D-VAC) in titanium alloy Ti6Al4V. Int J Adv Manuf Technol 117:1739–1764. 10.1007/s00170-021-07831-8

[46] Zhao Q, Sun Z, Guo B (2016) Material removal mechanism in ultrasonic vibration assisted polishing of micro cylindrical surface on SiC. Int J Mach Tools Manuf 103:28–39. 10.1016/j.ijmachtools.2016.01.003

[47] Ban X, Zhu J, Sun G, Han S, Duan T, Wang N (2024) Molecular simulation of ultrasonic assisted diamond grit scratching 4H-SiC single-crystal. Tribol Int 192:109–330. 10.1016/j.triboint.2024.109330

[48] Yu W, Chen J, An Q, Ming W, Chen M (2023) Investigations on the effect of ultrasonic vibration on fibre fracture and removal mechanism in cutting of fibre reinforced silicon carbide ceramic matrix composites. J Manuf Process 94:359–373. 10.1016/j.jmapro.2023.03.049

[49] Chen Y, Pan L, Yin Z, Wu Y (2024) Effects of ultrasonic vibration-assisted machining methods on the surface polishing of silicon carbide. J Mater Sci 59:7700–7715. 10.1007/s10853-024-09661-x

[50] Liu C, Wang WH, Xiong YF, Xiong B, Li LW (2023) Experimental investigation on tool wear in ultrasonic vibration-assisted turning of SiC_f_/SiC ceramic matrix composite. Int J Adv Manuf Technol 125:3081–3101. 10.1007/s00170-023-10896-2

[51] Guo Y, Lee YJ, Zhang Y, Wang H (2022) Magneto-plasticity in micro-cutting of single-crystal copper. J Mater Sci Technol 124:121–134. 10.1016/j.jmst.2022.03.003

[52] Xiao JF, Guo F, Zhang C, Chen X, Zhang JG, Xu JF (2022) Experimental investigation for ultra-precision cutting of nickel based superalloy with the assistance of magnetic field. Sci China Technol Sci 65:2170–2177. 10.1007/s11431-022-2150-4

[53] Mansori ME, Iordache V, Seitier P, Paulmier D (2004) Improving surface wearing of tools by magnetization when cutting dry. Surf Coat Technol 188:566–571. 10.1016/j.surfcoat.2004.07.037

[54] Khalil AK, Yip WS, To S (2022) Theoretical and experimental investigations of magnetic field assisted ultra-precision machining of titanium alloys. J Mater Process Technol 300:117429. 10.1016/j.jmatprotec.2021.117429

[55] Li D, Yip WS, Cao H, Zhang H, Tang YM, To S (2023) Chatter suppression in diamond turning using magnetic field assistance. J Mater Process Technol 321:118150. 10.1016/j.jmatprotec.2023.118150

[56] Xu Z, Liu L, He Z, Tian D, Hartmaier A, Zhang J, Luo X, Rommel M, Nordlund K, Zhang G, Fang F (2020) Nanocutting mechanism of 6H-SiC investigated by scanning electron microscope online observation and stress-assisted and ion implant-assisted approaches. Int J Adv Manuf Technol 106:3869–3880. 10.1007/s00170-019-04886-6

[57] Kang Q, Fang XD, Wu C, Verma P, Sun H, Tian B, Zhao LB, Wang SL, Zhu N, Maeda R, Jiang ZD (2022) Mechanical properties and indentation-induced phase transformation in 4H–SiC implanted by hydrogen ions. Ceram Int 48:15334–15347. 10.1016/j.ceramint.2022.02.067

[58] Fan YX, Xu ZW, Song Y, Dong B, Xue ZF, Liu B, Liu L, Tian DY (2021) Nano material removal mechanism of 4H-SiC in ion implantation-assisted machining. Comput Mater Sci 200:110837. 10.1016/j.commatsci.2021.110837

[59] Dai HF, Hu Y, Wu WL, Yue HX, Meng XS, Li P, Duan HG (2021) Molecular dynamics simulation of ultra-precision machining 3 C-SiC assisted by ion implantation. J Manuf Process 69:398–411. 10.1016/j.jmapro.2021.07.055

[60] Tan Y, Yip WS, Zhao T, To S, Zhao Z (2024) Subsurface damage and brittle fracture suppression of monocrystalline germanium in ultra-precision machining by multiple ion implantation surface modification. J Mater Process Technol 334:118640. 10.1016/j.jmatprotec.2024.118640

[61] Zhang LF, Ren CZ, Ji CH, Wang ZQ, Chen G (2016) Effect of fiber orientations on surface grinding process of unidirectional C/SiC composites. Appl Surf Sci 366:424–431. 10.1016/j.apsusc.2016.01.142

[62] Tashiro T, Fujiwara J, Takenaka Y (2007) Grinding of C/C-SiC composite in dry method, in: Proceedings of the 11th International Conference on Precision Engineering (ICPE), Springer, London, pp. 351–352

[63] Coroado J, Ganguly S, Williams S, Suder W, Meco S, Pardal G (2022) Comparison of continuous and pulsed wave lasers in keyhole welding of stainless-steel to aluminium. Int J Adv Manuf Technol 119:367–387. 10.1007/s00170-021-08226-5

[64] Liu JL, Chen X, Chen YQ, Cui YH, Guo SR, Wu XW, Cui LJ (2024) Laser cleaning of RTV coating on the insulator surface by using millisecond pulse lasers. Appl Opt 63:2271–2278. 10.1364/AO.515713 38568582 10.1364/AO.515713

[65] Zhang N, Li L, Yu A (2022) Laser technology for precise material machining applications. Photonics Views 19:46–50. 10.1002/phvs.202200050

[66] Chen JL, Lu XZ, Wen QL, Jiang F, Lu J, Lei DJ, Pan YC (2021) Review on laser-induced etching processing technology for transparent hard and brittle materials. Int J Adv Manuf Technol 117:2545–2564. 10.1007/s00170-021-07853-2

[67] Bernard B, Matylitsky V (2017) Laser micro-machining strategies for transparent brittle materials using ultrashort pulsed lasers, Laser-based Micro-and nano processing XI. SPIE 10092:2252105. 10.1117/12.2252105

[68] Erdenechimeg K, Jeong HI, Lee CM (2019) A study on the laser-assisted machining of carbon fiber reinforced silicon carbide. Materials 12:2061. 10.3390/ma12132061 31252524 10.3390/ma12132061 PMC6651644

[69] Shahinian H, Navare J, Zaytsev D (2019) Microlaser assisted diamond turning of precision silicon optics. Opt Eng 58:092607. 10.1117/1.OE.58.9.092607

[70] You KY, Yan GP, Luo XC, Gilchrist MD, Fang FZ (2020) Advances in laser assisted machining of hard and brittle materials. J Manuf Process 58:677–692. 10.1016/j.jmapro.2020.08.034

[71] Bharat N, Bose PSC (2021) An overview on machinability of hard to cut materials using laser assisted machining. Mater Today Proc 43:665–672. 10.1016/j.matpr.2020.12.587

[72] Kumar M, Melkote S, Lahoti G (2011) Laser-assisted microgrinding of ceramics. CIRP Ann 60:367–370. 10.1016/j.cirp.2011.03.121

[73] Chang CW, Kuo CP (2007) An investigation of laser-assisted machining of Al2O3 ceramics planning. Int J Mach Tools Manuf 47:452–461. 10.1016/j.ijmachtools.2006.06.010

[74] Zhou L, Zhang Z, Lin Z, Huang P, Jiao H, Zhang G, Huang Y, Zhou J, Long Y (2024) Study on the control and mechanism of balance between heat and cold for spray-mist-assisted laser processing of CFRP. Opt Laser Technol 174:110682. 10.1016/j.optlastec.2024.110682

[75] Duan WQ, Dong X, Wang KD, Fan ZJ, Mei XS, Wang WJ, Lv J (2016) Effect of temporally modulated pulse on reducing recast layer in laser drilling. Int J Adv Manuf Technol 87:1641–1652. 10.1007/s00170-016-8362-5

[76] Wang ZD, Zhai CT, Yu P, Tian JW, Nie XY, Xu JK (2020) Simulation and experimental study on laser assisted micro cutting of aluminum based silicon carbide. Modular Mach Tool Automatic Manuf Technique 10:59–63. 10.13462/j.cnki.mmtamt.2020.10.015

[77] Cao C, Zhao YG, Zhang GG, Li ZH, Zhao C, Yu HL, Zhao DD, Zhang HY, Dai D (2024) Experimental study of plastic cutting in laser-assisted machining of SiC ceramics. Opt Laser Technol 169:110098. 10.1016/j.optlastec.2023.110098

[78] Dai D, Cai YK, Zhao YG, Aslam J, Tang Y, Liang XL, Liu ZQ (2025) Study on the plastic removal behaviour of SiC ceramic materials in laser-assisted high-temperature turning. Opt Laser Technol 192:113566. 10.1016/j.optlastec.2025.113566

[79] Zhang JG, Fu YF, Chen X, Shen ZF, Zhang JJ, Xiao JF, Xu JF (2023) Investigation of the material removal process in in-situ laser-assisted diamond cutting of reaction-bonded silicon carbide. J Eur Ceram Soc 43:2354–2365. 10.1016/j.jeurceramsoc.2023.01.011

[80] Dai D, Zhao YG, Cao C, Dong RC, Zhang HY, Liu Q, Song Z, Zhang XJY, Zheng ZL, Zhao C (2022) Experimental investigation on process parameters during laser-assisted turning of SiC ceramics based on orthogonal method and response surface methodology. Materials 15:4889. 10.3390/ma15144889 35888357 10.3390/ma15144889 PMC9318182

[81] Cao C, Zhao YG, Meng JB, Dai D, Liu Q, Liu GX, Zhou HA, Song Z, Zhang HY, Zhang XJY (2023) Experimental study of the thermal effects and processing in CW laser-assisted turning of SiC ceramics. Int J Adv Manuf Technol 125:4467–4483. 10.1007/s00170-023-10945-w

[82] Viskup R (2016) High Energy and Short Pulse Lasers. Intech Open, Rijeka. 10.5772/61628

[83] Tadavani SA, Razavi RS, Vafaei R (2017) Pulsed laser-assisted machining of inconel 718 superalloy. Opt Laser Technol 87:72–78. 10.1016/j.optlastec.2016.07.020

[84] Liu YM, Liang GY, Gu Y, Lin JQ, Fu B, Gao TY, Zhao JX, Luan YL (2025) Study on PCD tool wear in pulsed laser assisted turning Al-50wt% Si alloy. Mater Today Commun 42:111181. 10.1016/j.mtcomm.2024.111181

[85] Guo MQ, Lu MM, Lin JQ, Zhou XQ (2025) Modeling and experimental study on cutting forces during pulsed laser-assisted fast tool servo turning free-form glass-ceramics. Ceram Int 51:6641–6653. 10.1016/j.ceramint.2024.12.108

[86] Paknejad M, Azarhoushang B, Zahedi A, Khakrangin M, Bösinger R, Hojati F (2024) Laser-assisted surface grinding of innovative superhard SiC-bonded diamond (DSiC) materials. Ceram Int 50:18391–18407. 10.1016/j.ceramint.2024.02.323

[87] Xiao GJ, Liu ZY, Geng YQ, He Y, Li C, Lin OC, Zhu SW (2023) Enhancing machinability of Ti6Al4V by ultrashort-pulse laser-induced modification assisted grinding. Int J Adv Manuf Technol 125:4601–4620. 10.1007/s00170-023-11073-1

[88] Ion JC (2005) Laser processing of engineering materials: principles, procedure and industrial application. Butterworth-Heinemann, Oxford, pp 41–103. 10.1016/B978-075066079-2/50006-4

[89] Wang C, Kurokawa S, Doi T, Yuan J, Sano Y, Aida H, Zhang K, Deng Q (2017) The polishing effect of SiC substrates in femtosecond laser irradiation assisted chemical mechanical polishing (CMP). ECS J Solid State Sci Technol 6:P105. 10.1149/2.0041704jss

[90] Gao B, Guo D, Zhang X, Chen GP, Pan GS (2021) Picosecond laser-assisted chemical mechanical polishing (CMP): aiming at the Si-face of single-crystal 6H-SiC wafer. ECS J Solid State Sci Technol 10:044008. 10.1149/2162-8777/abf726

[91] Liu HX, Li ZP, Zhang P, Zuo DW, Xie WK (2024) Study of damage mechanism on single crystal 4H-SiC surface layer by picosecond laser modification (PLM). Appl Surf Sci 672:160722. 10.1016/j.apsusc.2024.160722

